# The MabZIP5–MaMYB69 module cooperates with MaERF55 to modulate banana fruit ripening via cell wall degradation

**DOI:** 10.1093/hr/uhaf275

**Published:** 2025-10-17

**Authors:** Fan Liu, Xueli Sun, Jianzhu Cao, Ou Sheng, Tongxin Dou, Qiaosong Yang, Chunhua Hu, Guiming Deng, Weidi He, Huijun Gao, Tao Dong, Chunyu Li, Yaoyao Li, Cancan Liu, Ganjun Yi, Fangcheng Bi

**Affiliations:** Key Laboratory of South Subtropical Fruit Biology and Genetic Resource Utilization (Ministry of Agriculture and Rural Affairs), Guangdong Provincial Key Laboratory of Science and Technology Research on Fruit Tree, Institute of Fruit Tree Research, Guangdong Academy of Agricultural Sciences, Guangzhou 510640, China; College of Life Sciences, South China Agricultural University, Guangzhou 510642, China; Key Laboratory of South Subtropical Fruit Biology and Genetic Resource Utilization (Ministry of Agriculture and Rural Affairs), Guangdong Provincial Key Laboratory of Science and Technology Research on Fruit Tree, Institute of Fruit Tree Research, Guangdong Academy of Agricultural Sciences, Guangzhou 510640, China; Key Laboratory of South Subtropical Fruit Biology and Genetic Resource Utilization (Ministry of Agriculture and Rural Affairs), Guangdong Provincial Key Laboratory of Science and Technology Research on Fruit Tree, Institute of Fruit Tree Research, Guangdong Academy of Agricultural Sciences, Guangzhou 510640, China; College of Life Sciences, South China Agricultural University, Guangzhou 510642, China; Key Laboratory of South Subtropical Fruit Biology and Genetic Resource Utilization (Ministry of Agriculture and Rural Affairs), Guangdong Provincial Key Laboratory of Science and Technology Research on Fruit Tree, Institute of Fruit Tree Research, Guangdong Academy of Agricultural Sciences, Guangzhou 510640, China; Key Laboratory of South Subtropical Fruit Biology and Genetic Resource Utilization (Ministry of Agriculture and Rural Affairs), Guangdong Provincial Key Laboratory of Science and Technology Research on Fruit Tree, Institute of Fruit Tree Research, Guangdong Academy of Agricultural Sciences, Guangzhou 510640, China; Key Laboratory of South Subtropical Fruit Biology and Genetic Resource Utilization (Ministry of Agriculture and Rural Affairs), Guangdong Provincial Key Laboratory of Science and Technology Research on Fruit Tree, Institute of Fruit Tree Research, Guangdong Academy of Agricultural Sciences, Guangzhou 510640, China; Key Laboratory of South Subtropical Fruit Biology and Genetic Resource Utilization (Ministry of Agriculture and Rural Affairs), Guangdong Provincial Key Laboratory of Science and Technology Research on Fruit Tree, Institute of Fruit Tree Research, Guangdong Academy of Agricultural Sciences, Guangzhou 510640, China; Key Laboratory of South Subtropical Fruit Biology and Genetic Resource Utilization (Ministry of Agriculture and Rural Affairs), Guangdong Provincial Key Laboratory of Science and Technology Research on Fruit Tree, Institute of Fruit Tree Research, Guangdong Academy of Agricultural Sciences, Guangzhou 510640, China; Key Laboratory of South Subtropical Fruit Biology and Genetic Resource Utilization (Ministry of Agriculture and Rural Affairs), Guangdong Provincial Key Laboratory of Science and Technology Research on Fruit Tree, Institute of Fruit Tree Research, Guangdong Academy of Agricultural Sciences, Guangzhou 510640, China; Key Laboratory of South Subtropical Fruit Biology and Genetic Resource Utilization (Ministry of Agriculture and Rural Affairs), Guangdong Provincial Key Laboratory of Science and Technology Research on Fruit Tree, Institute of Fruit Tree Research, Guangdong Academy of Agricultural Sciences, Guangzhou 510640, China; Key Laboratory of South Subtropical Fruit Biology and Genetic Resource Utilization (Ministry of Agriculture and Rural Affairs), Guangdong Provincial Key Laboratory of Science and Technology Research on Fruit Tree, Institute of Fruit Tree Research, Guangdong Academy of Agricultural Sciences, Guangzhou 510640, China; Key Laboratory of South Subtropical Fruit Biology and Genetic Resource Utilization (Ministry of Agriculture and Rural Affairs), Guangdong Provincial Key Laboratory of Science and Technology Research on Fruit Tree, Institute of Fruit Tree Research, Guangdong Academy of Agricultural Sciences, Guangzhou 510640, China; Key Laboratory of South Subtropical Fruit Biology and Genetic Resource Utilization (Ministry of Agriculture and Rural Affairs), Guangdong Provincial Key Laboratory of Science and Technology Research on Fruit Tree, Institute of Fruit Tree Research, Guangdong Academy of Agricultural Sciences, Guangzhou 510640, China; Key Laboratory of South Subtropical Fruit Biology and Genetic Resource Utilization (Ministry of Agriculture and Rural Affairs), Guangdong Provincial Key Laboratory of Science and Technology Research on Fruit Tree, Institute of Fruit Tree Research, Guangdong Academy of Agricultural Sciences, Guangzhou 510640, China; Key Laboratory of South Subtropical Fruit Biology and Genetic Resource Utilization (Ministry of Agriculture and Rural Affairs), Guangdong Provincial Key Laboratory of Science and Technology Research on Fruit Tree, Institute of Fruit Tree Research, Guangdong Academy of Agricultural Sciences, Guangzhou 510640, China; Key Laboratory of South Subtropical Fruit Biology and Genetic Resource Utilization (Ministry of Agriculture and Rural Affairs), Guangdong Provincial Key Laboratory of Science and Technology Research on Fruit Tree, Institute of Fruit Tree Research, Guangdong Academy of Agricultural Sciences, Guangzhou 510640, China

## Abstract

The MYB protein family comprises numerous transcription factors with important functions in various biological processes in plants; however, their role in modulating banana fruit ripening has been rarely investigated. In this study, we identified an R2R3-type MYB gene, *MaMYB69*, which promotes fruit ripening by directly modulating cell wall degradation. The expression of *MaMYB69*, which encodes a nucleus-localized transcription activator, was induced by ethylene and upregulated during banana ripening. MaMYB69 activated the expression of *MaPE*, *MaPL1*, *MaGAL*, and *MaPG3* by directly targeting their promoters. *MaMYB69* overexpression in tomato and banana accelerated fruit ripening and stimulated cell wall-modifying gene expression. Moreover, MaMYB69 interacted with MaERF55 and strengthened its transactivation activity through downstream target genes. MabZIP5, an aroma biosynthesis regulator, acted directly upstream of *MaMYB69*, enhancing its transcription. MaMYB69 also formed a homodimer with itself and activated its expression. Collectively, our results show that MaMYB69 is pivotal in controlling banana ripening and softening and improve our understanding of the regulatory cascades involved in fruit ripening. MaMYB69 may serve as a potential target for improving fruit quality and extending the shelf life.

## Introduction

Fruit ripening determines the final quality and economic value of fruit. It is a highly intricate process that occurs during the terminal phase of fruit development and involves a series of coordinated physiological and biochemical transformations, including color alterations mediated by chlorophyll degradation and pigment accumulation; flavor improvement through sugar, organic acid, and volatile aromatic compound biosynthesis; and fruit softening driven by the structural reorganization of polysaccharide components [1, 2]. Fruit softening is a prominent characteristic of fruit ripening; however, it is a double-edged sword. Although moderate softening attracts consumers, excessive softening reduces postharvest transportability and storability, shortening the shelf life and causing significant postharvest losses [3, 4]. Fruit softening is closely linked to cell wall component disassembly and cell structure loss [5]. During fruit ripening, cell wall polysaccharides (e.g. pectin, cellulose, hemicellulose) undergo solubilization and depolymerization, which reduce intercellular adhesion, enlarge cell space, and decrease cell wall density and thickness [3, 6]. These processes are facilitated by cell wall degradation-related enzymes and proteins, such as polygalacturonases (PGs), pectate lyases (PLs), β-galactosidases (β-Gals), pectin methylesterases (PMEs), xyloglucan endotransglucosylases/hydrolases (XETs/XTHs), and expansins (EXPs) [4, 7]. Altering the expression level of the genes for cell wall enzymes significantly influences fruit ripening and softening [8, 9]. In tomato, the silencing or knockout of *SlPG* [10], *SlPL* [11], *SlPME2* [4], *SlPMEU1 ,*[4], or *SlTBG4* [8] enhances fruit firmness and delays fruit softening. In strawberry, the loss of *FaPG1* function [12] and antisense silencing of *FvPLA* [9] or *FaβGal4* [13] attenuate the softening and extend the shelf life of fruit. Knockdown or knockout of more than one gene may result in larger reductions in softening. Enzymes SlPG2a and SlPL act additively, and simultaneous knockout of these genes inhibits fruit softening, enhances fruit disease resistance, and does not affect fruit quality traits [14]. Although the link between cell wall degradation genes and fruit softening is well established, our understanding of the transcriptional mechanisms governing these genes during fruit ripening and softening remain poorly understood.

Many transcription factors (TFs) participate in fruit softening by triggering or repressing the expression of cell wall degradation genes. In tomato, SlLOB1 promotes fruit softening by governing *SlEXP1*, *SlCEL2*, *SlXY*, *SlPL*, *SlTBL*, *SlE6*, and *SlAGP2* [15]. Similarly, SlNAC4 accelerates fruit ripening and softening by directly binding to the promoters of *SlEXP1* and *SlCEL2* and activating their expression [16]. In apple, *MdZF-HD11* promotes postharvest softening by inducing activation of *Mdβ-GAL18* [17]. Transcriptional activators AcNAC1 and AcNAC2 participate in kiwifruit softening by enhancing the transcription of *AcXTH1* and *AcXTH2* [18]. Moreover, the basic helix–loop–helix (bHLH) TF AaBIM1 delays kiwiberry softening by repressing the expression of cell wall metabolism-associated genes *AaPG1*, *AaXTH28*, *AaPME1,* and *AaPME2* [19]. In sweet cherry, PavDof2 and PavDof15 attenuate fruit softening by repressing the transcription of cell wall degradation genes through direct promoter binding, whereas PavDof6 promotes fruit softening by activating their transcription [6]. In banana, MaWRKY49 modulates fruit softening by interacting with the promoters of *MaPL3* and *MaPL11* and activating their transcription [20]. MabHLH28 accelerates fruit softening by interacting with MaWRKY49 and MaWRK111 to form protein complexes that strengthen the expression of cell wall degradation genes [21]. MaNAC154 suppresses the transcription of cell wall degradation genes and recruits MaHDA6, enhancing the inhibitory capacity of MaNAC154 [22]. Although some TFs exert functions in fruit ripening and softening, additional modulators involved in the ripening regulatory network need to be explored.

MYB TFs are among the most widely distributed TFs in eukaryotes. MYB proteins feature a highly conserved N-terminal MYB domain (typically 50–53 amino acids) that mediates binding to a specific motif in the promoter, such as ACC(A/T)(A/C/T)(A/C/T) and (C/T)AAC(G/T)G [23]. The C-terminus of MYBs typically contains a transcriptional regulatory region enriched in acidic amino acids, which governs the transcription of target genes [24]. Based on variations in MYB domain repeats, MYB proteins are classified into four subfamilies: 1R-MYB, 3R-MYB, 4R-MYB, and R2R3-MYB [23, 24]. Among MYB subfamilies, R2R3-MYB is the largest and most well studied. R2R3-MYB TFs are pivotal for controlling fruit ripening, appearance, and intrinsic quality attributes. In tomato, SlMYB70 negatively controls fruit ripening by repressing the expression of *SlACO3* and *SlACS2* [25]. SlMYB75 stimulates anthocyanin biosynthesis and aroma volatile production [26]. In sweet cherry, PavMYB10.1 promotes anthocyanin biosynthesis by assembling with PavbHLH and PavWD40 into an MBW transcriptional complex that directly targets the promoters of PavANS and PavUFGT [27]. In banana, MaMYB3 delays fruit ripening by suppressing starch degradation through the transcriptional repression of *MabHLH6* and downstream starch catabolism genes [28]. MaMYB60 degrades chlorophyll by directly stimulating the transcription of chlorophyll catabolic genes; in contrast, MaMYB60 is degraded by MaBAH1-mediated E3 ubiquitination under high temperatures, resulting in green ripening in banana [29]. However, limited research is available on the regulatory relationship between MYBs and fruit texture and softening. *FvMYB79* overexpression reduces strawberry fruit firmness and accelerates ripening by increasing the transcriptional level of *FvPME38* [30]. Similarly, transient *MdMYB44* overexpression enhances apple crispness by directly activating the expression of *MdMPE3* [31]. Conversely, transient *MaMYB4* overexpression delays banana ripening by suppressing ethylene production and cell wall disassembly [32]. Collectively, these findings pave the way for advanced research into the roles of MYB TFs in fruit softening.

Banana ranks among the world’s most popular tropical fruits and constitutes the fourth most vital food crop after rice, wheat, and maize. It is highly favored by consumers owing to its pleasant flavor, distinctive aroma, and nutritional richness, including carbohydrates, vitamins, trace elements, and dietary fiber [33]. Banana is generally harvested at 75% maturity and treated with ethylene gas/ethephon to ripen before marketing. Once ripening is initiated, bananas turn yellow and rapidly soften, with a short shelf life of approximately a week, which adversely impacts fruit quality and limits its commodity value [34]. Each year, 25%–50% of bananas are lost after harvest, with rapid softening representing a major contributing factor [34, 35]. Therefore, elucidating the transcriptional control mechanisms underlying fruit softening is essential for reducing postharvest losses.

In this study, we demonstrate that MaMYB69 (Ma04_g16770) functions as a transcriptional activator in banana, upregulating the expression of *MaPE*, *MaPL1*, *MaGAL*, and *MaPG3* through direct promoter binding. *MaMYB69* overexpression in tomato fruit significantly promoted fruit ripening and softening. Moreover, MaMYB69 interacted with MaERF55 to form a heterodimer, which enhanced the transcriptional activation of MaMYB69-controlled target genes. MabZIP5, an aroma biosynthesis regulator, functioned as an upstream activator of *MaMYB69*. Overall, this investigation reveals a vital regulatory mechanism for banana cell wall disassembly during ripening and lays a theoretical foundation for improving fruit quality and prolonging postharvest longevity.

## Results

### Determination and characterization of MYB TFs involved in banana fruit ripening

**Figure 1:**
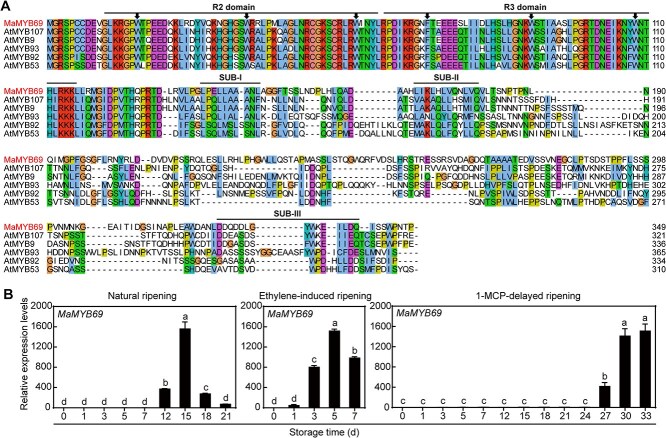
Sequence and expression patterns of MaMYB69. (A) Alignment of the MaMYB69 amino acid sequence with *Arabidopsis* MYBs. The conserved R2 and R3 MYB domains and three C-terminal motifs (SUB-I, SUB-II, and SUB-III) are indicated by lines above them. Arrows denote the conserved amino acids tryptophan (W) and phenylalanine (F). . (B) Expression patterns of *MaMYB69* in pulp tissue. Results are presented as mean ± SD from three independent replicates. Statistically significant differences (*P* < 0.05) identified by one-way ANOVA are indicated by distinct lowercase letters.

Based on our previous transcriptomic data for banana fruit ripening, 11 activated genes belonging to the MYB gene family during fruit ripening were retrieved [36, 37]. The expression patterns of these transcripts are presented in Fig. S1. Of these transcripts, the expression of *MaMYB69*, *MaMYB151*, and *MaMYB177* was significantly induced by ethylene and inhibited by 1-methylcyclopropene (1-MCP), suggesting that MaMYB69, MaMYB151, and MaMYB177 may be associated with fruit ripening. The gene coding sequences were cloned and analyzed to dissect the characterization and function of MaMYB69, MaMYB151, and MaMYB177. The coding MaMYB69, MaMYB151, and MaMYB177 proteins were 348, 357, and 274 amino acid (aa) residues in length, with predicted molecular weights (MWs) of 38705.55, 39298.39, and 30623.37 Da, respectively. Containing the typical conserved R2R3-type domain, these proteins were classified as R2R3 MYB TFs. Phylogenetic analysis revealed that MaMYB69 belonged to subgroup 10, and MaMYB151 and MaMYB177 belonged to subgroup 21 (Fig. S2). MaMYB69 shared the highest similarity with AtMYB9, followed by AtMYB107, and contained three conserved motifs: SUB-I, SUB-II, and SUB-III (Fig. 1A). MaMYB151 and MaMYB177 were closely related to AtMYB56 and contained an FxDFL motif in the C-terminal region (Fig. S3A). We analyzed the expression trends in *MaMYB69*, *MaMYB151*, and *MaMYB177* under natural, ethylene, and 1-MCP treatments using quantitative real-time polymerase chain reaction (qRT-PCR) to identify the accuracy of the transcriptomic data. The results were consistent with the transcriptomic data, and the transcription of these genes was significantly induced during fruit ripening (Fig. 1B and Fig. S3B), indicating that these genes play a vital role in banana ripening.

### MaMYB69 is a transcriptional activator localized in the nucleus

**Figure 2:**
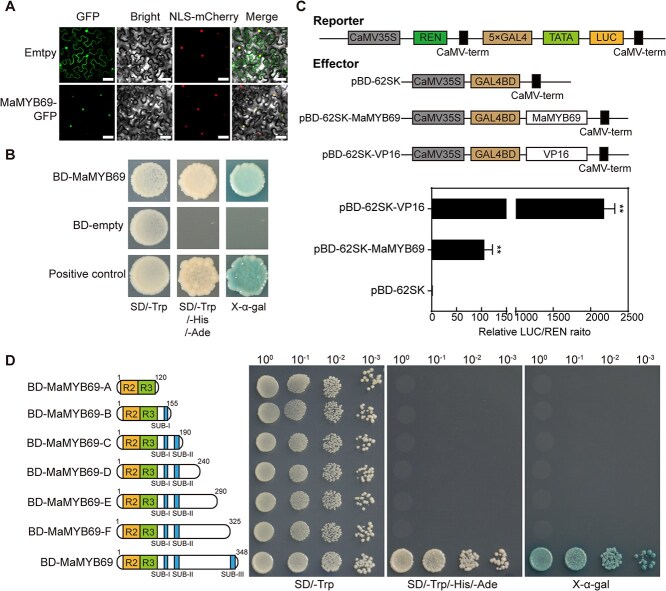
Subcellular localization and transcriptional activation analysis of MaMYB69. (A) Subcellular localization of MaMYB69 in plant cells. Empty-GFP and MaMYB69-GFP plasmids were transiently expressed in tobacco leaves via *Agrobacterium*-mediated transformation. Empty-GFP and NLS-mCherry vectors served as the negative control and nuclear marker, respectively. Bar = 40 μm. (B) Transcriptional activation capacity of MaMYB69 in yeast cells. The fusion construct pGBKT7-MaMYB69 (BD-MaMYB69), negative control pGBKT7 (BD-empty), and positive control pGBKT7-p53/pGADT7-T-antigen were separately introduced into Y2HGold yeast cells. Transformed cells were plated on SD/−Trp, SD/−Trp/-His/−Ade, and SD/−Trp/-His/−Ade/+X-α-gal media. (C) Transcriptional activity analysis of MaMYB69 using a DLR assay system in rice protoplasts. The upper panel shows a schematic of the reporter and effector constructs. pBD-62SK-VP16 and pBD-62SK served as positive and negative controls, respectively. The LUC/REN ratio for the empty pBD-62SK and reporter vector combination is defined as 1. Symbol (**) marks significant differences at *P* < 0.01 (Student’s *t*-test). (D) Transactivation assay of intact or different truncated MaMYB69 proteins in yeast. Different lengths of MaMYB69 were fused to the GAL4 DNA-BD and transformed into the Y2HGold yeast cells. The growth and coloration of yeast cells on X-α-gal-supplemented SD/−Trp/-His/−Ade medium indicate transcriptional activation mediated by each protein.

MYB TFs are typically nuclear proteins. To study the subcellular localization of these MaMYB genes, we generated 35S-MaMYB69-GFP, 35S-MaMYB151-GFP, and 35S-MaMYB177-GFP recombinant plasmids and transiently coexpressed them in tobacco leaves with NLS-mCherry plasmid (nuclear marker). The fluorescent signals of the three fusion proteins were observed in the nucleus, which clearly overlapped with the fluorescent signal of the nuclear marker, whereas the green fluorescent protein (GFP) signal of the empty vector appeared in both the nucleus and cytoplasm (Fig. 2A and Fig. S4A). These findings support that these genes function as TFs.

To determine the transcriptional activation ability of MaMYB69, MaMYB151, and MaMYB177, we generated the recombinant plasmids by fusing their coding sequences (CDS) to the GAL4 DNA-binding domain (BD) in the pGBKT7 vector and transforming them into the yeast two-hybrid (Y2H) Gold yeast cells. The yeast cells carrying BD-MaMYB69 and BD-MaMYB177 grew well and appeared blue on the SD-WHA medium (SD minimal medium lacking Trp, His, and Ade) in the presence of X-α-Gal (Fig. 2B and Fig. S4B), whereas cells carrying BD-MaMYB151 grew only slightly on the same medium, suggesting that MaMYB69 and MaMYB177 have strong activation activity in yeast. We further performed the dual-luciferase reporter (DLR) assay to confirm the results in rice protoplasts. Compared with the control, pBD-62SK-MaMYB69 showed an evident increase in luciferase (LUC) reporter gene activity (Fig. 2C and Fig. S4C), whereas pBD-62SK-MaMYB151 and pBD-62SK-MaMYB177 had no effect on LUC activity. This result indicates that only MaMYB69 acts as a transcriptional activator in plants. The transactivation of MaMYB69 in yeast and plants suggests that it possesses the typical features of TFs. Thus, we selected MaMYB69 for subsequent analyses.

To further determine which region of MaMYB69 contributes to its transactivation ability, we amplified a series of truncated MaMYB69 sequences and ligated it into the pGBKT7 plasmid. Yeast cells containing BD-MaMYB69-F could not grow on the SD-WHA medium (Fig. 2D), whereas BD-MaMYB69 grew under the same conditions, indicating that the activation domain of MaMYB69 existed within the C-terminal region (326–348 aa). These observations reveal that MaMYB69 acts as an important transcriptional activator during banana ripening.

### MaMYB69 activates cell wall-modifying genes and itself

**Figure 3:**
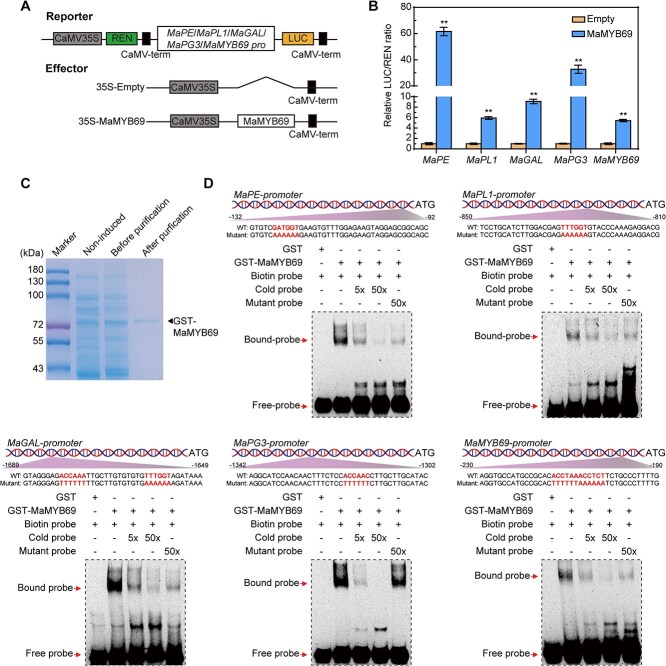
MaMYB69-mediated direct regulation of *MaPE*, *MaPL1*, *MaGAL*, *MaPG3*, and *MaMYB69* in banana. (A) Schematic representation of the LUC reporter and effector constructs. (B) MaMYB69 activates the expression of *MaPE*, *MaPL1*, *MaGAL*, *MaPG3*, and *MaMYB69*. LUC activity values were normalized to REN. The relative LUC/REN ratio indicates the activity of each reporter activated by MaMYB69. The LUC/REN ratio for the empty vector and reporter combination is defined as 1. Double asterisks (**) mark significant differences at *P* < 0.01 (Student’s *t*-test). (C) Purified recombinant GST-MaMYB69 protein visualized by SDS-PAGE. (D) EMSA showing the binding of MaMYB69 to the promoters of *MaPE*, *MaPL1*, *MaGAL*, *MaPG3*, and *MaMYB69*. The upper panel indicates the sequences and positions of the probes on the promoters. The bold letters in the probe sequence represent the MaMYB69 binding sites. Symbols + and – indicate probe/protein inclusion and exclusion, respectively. Competition experiments employed five-fold and 50-fold molar excesses of unlabeled probes, along with a 50-fold molar excess of mutant probes.

Fruit softening is modulated by various cell wall metabolism-modifying enzymes and is the major factor affecting postharvest losses. To determine whether MaMYB69 influences the expression of cell wall-modifying genes, four significantly upregulated genes associated with cell wall disassembly in fruit ripening were selected as potential targets of MaMYB69 (Fig. S5). We generated five reporter vectors containing the LUC reporter gene controlled by the promoters of these genes and an effector vector including CaMV 35S promoter-driven MaMYB69 expression cassette (Fig. 3A). The DLR assay indicated that MaMYB69 significantly enhanced the activities of *MaPE*, *MaPL1*, *MaGAL*, *MaPG3*, and *MaMYB69* promoters, evidenced by the relatively higher LUC/REN ratios than those in the controls (Fig. 3B). This result supports that MaMYB69 activates the transcription of *MaPE*, *MaPL1*, *MaGAL*, *MaPG3*, and itself.

R2R3-MYBs generally control the expression of their target genes by interacting with MYB-core (YNGTTR) and AC-box (ACCWHH) motifs within the promoters [38]. The DNA-binding motif analysis of MaMYB69 from the footprintDB and JASPAR databases revealed that the binding motif of MaMYB69 has a higher similarity to that of AtMYB107 (MA2007.1), which strongly resembles the AC-box (ACCWHH). Scanning the promoters of *MaPE*, *MaPL1*, *MaGAL*, *MaPG3*, and *MaMYB69* using the FIMO tool in MEME revealed that at least one predicted binding motif was found in each promoter (Text S1). Electrophoretic mobility shift assay (EMSA) was performed to determine whether MaMYB69 directly binds to these promoter sequences. The recombinant GST-MaMYB69 protein was produced in *Escherichia coli* cells and purified (Fig. 3C), and the promoter fragments from *MaPE*, *MaPL1*, *MaGAL*, *MaPG3*, and *MaMYB69* were labelled with biotin. As illustrated in Fig. 3D, incubation of the GST-MaMYB69 protein with the biotin-labeled probe resulted in band shifts, whose intensity decreased with higher concentrations of cold probe but remained unaffected by the mutant probe. This evidence confirms that MaMYB69 directly binds to the promoters of *MaPE*, *MaPL1*, *MaGAL*, *MaPG3*, and *MaMYB69* and activates their transcription.

### Overexpression of *MaMYB69* in tomato promotes fruit ripening

**Figure 4:**
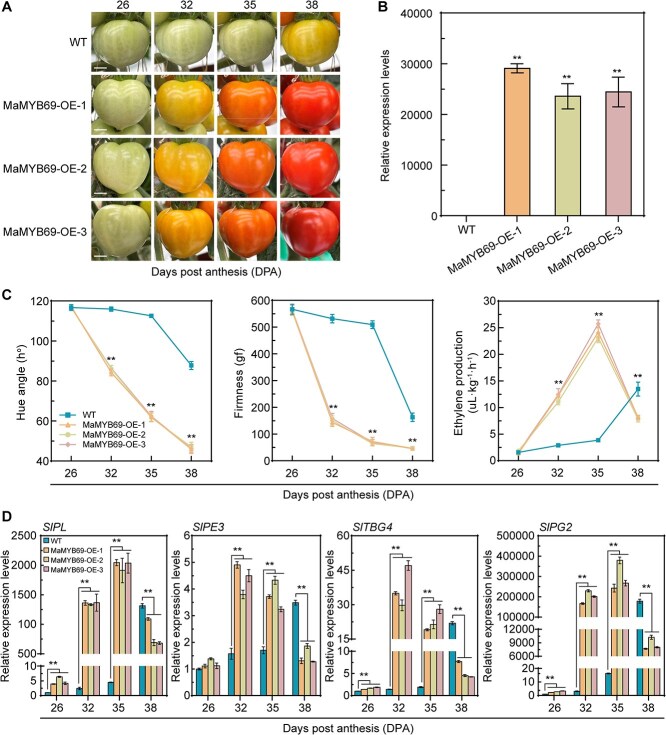
*MaMYB69* overexpression promotes tomato fruit ripening. (A) Phenotypic alterations of tomato fruit in WT and *MaMYB69* overexpression lines at 26, 32, 35, and 38 DPA. Bar: 0.5 cm. (B) Expression level of *MaMYB69* in fruit pericarp at 26 DPA examined via qRT-PCR. *SlUBI* was used as a reference gene. (C) Changes in tomato hue angle, fruit firmness, and ethylene production during tomato ripening. Data reflect the mean ± SD of three experimental repetitions. (D) Expression levels of *SlPL*, *SlPE3*, *SlTBG4*, and *SlPG2* in pericarp tissues analyzed by qRT-PCR and standardized with *SlUBI*. Values are expressed relative to WT at 26 DPA (set as 1). Data denote the mean ± SD of three independent experiments. Double asterisks (**) mark significant differences at *P* < 0.01 (Student’s *t*-test).

To further investigate the biological function of *MaMYB69* in fruit ripening, we generated *MaMYB69-*overexpressing tomatoes using ‘Micro-Tom’ variety (Fig. 4A). *MaMYB69* expression in transgenic tomato fruit was confirmed by qRT-PCR (Fig. 4B). We selected three independent transgenic lines for subsequent assays. Transgenic fruits overexpressing MaMYB69 exhibited accelerated ripening compared with the wild-type (WT) fruit (Fig. 4A). Compared with the WT fruit, the *MaMYB69*-OE transgenic fruit showed a visible phenotypic difference at 32 days post-anthesis (DPA). At this time, the *MaMYB69*-OE fruit entered the color-breaker stage, whereas the WT fruit remained in the mature green phase. The WT fruit initiated ripening at 38 DPA, whereas the *MaMYB69*-OE fruit reached the red-ripening stage at this time point. Compared with the WT fruit, the *MaMYB69*-OE fruit exhibited a rapid decline in firmness and hue angle starting at 32 DPA, and ethylene production peaked at 35 DPA (Fig. 4C). We further analyzed the transcription profiling of cell wall degradation genes *SlPL*, *SlPE3*, *SlTBG4*, and *SlPG2* during transgenic tomato fruit ripening. As presented in Fig. 4D, the transcription of *SlPL*, *SlPE3*, *SlTBG4*, and *SlPG2* was rapidly elevated in the *MaMYB69*-OE tomato fruit compared with the WT fruit, consistent with its accelerated ripening phenotype. Collectively, these observations indicate that *MaMYB69* overexpression in tomato fruit improves fruit ripening by activating the transcription of *SlPL*, *SlPE3*, *SlTBG4*, and *SlPG2*.

### Transient overexpression of *MaMYB69* in banana accelerates fruit ripening and softening

**Figure 5:**
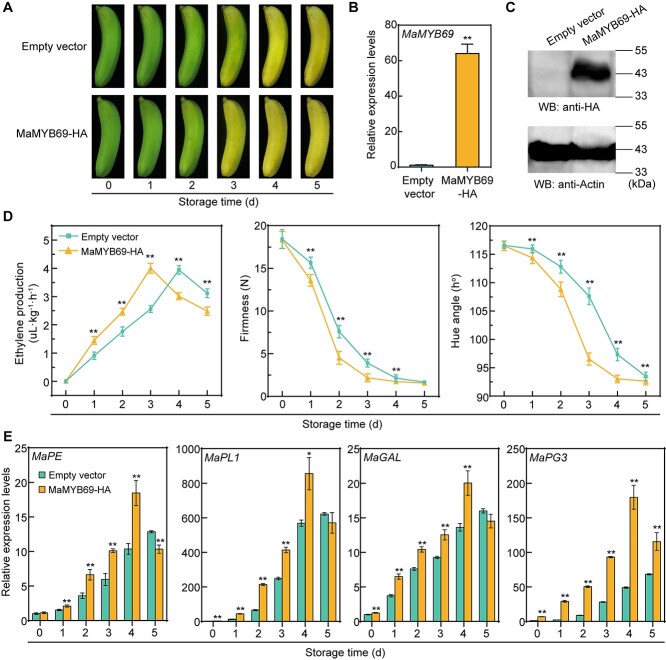
Transient overexpression of *MaMYB69* promotes banana fruit ripening and softening. (A) Phenotypic changes in the ripening of banana fruits transiently overexpressing *MaMYB69-HA* and empty vector control. (B) qRT-PCR and (C) western blot analyses confirmed the successful overexpression of MaMYB69 at the transcriptional and protein levels in banana fruit at 3 days. *MaCAC* gene and actin were used as the reference gene and loading control, respectively. (D) Changes in ethylene production, fruit firmness, and hue angle during ripening of *MaMYB69*-overexpressing and control banana fruits. (E) Relative expression levels of *MaPE*, *MaPL1*, *MaGAL*, and *MaPG3* in *MaMYB69*-overexpressing and control banana fruits during ripening. Data are presented as mean ± SD of three independent experiments. Asterisks denote significant differences between treatments (Student’s *t*-test, ^*^P < 0.05, ^**^P < 0.01).

To gain deeper insights into the role of *MaMYB69* in banana fruit ripening, we conducted transient overexpression of *MaMYB69* in banana fruits due to the difficulty of achieving stable transformation in this species. First, we transiently overexpressed *MaMYB69-HA* and the empty vector in mature green banana fruits via *Agrobacterium*-mediated infiltration, followed by ethylene treatment for ripening (Fig. 5A). Subsequently, qRT-PCR and immunoblotting analyses demonstrated that the gene expression and protein accumulation of MaMYB69 in the overexpression group exceeded those in the empty vector (control) group (Fig. 5B and C), suggesting successful *MaMYB69* overexpression. *MaMYB69*-overexpressing banana fruits exhibited a more pronounced yellowing phenotype than those infiltrated with the control bacteria, indicating that *MaMYB69* accelerated banana ripening (Fig. 5A). At the same time, *MaMYB69*-overexpressing banana exhibited higher ethylene production and lower fruit firmness and hue angle than the control (Fig. 5C), consistent with the observed phenotypic changes. Moreover, the expression of MaMYB69 target genes, such as *MaPE*, *MaPL1*, *MaGAL*, and *MaPG3*, was markedly induced in the *MaMYB69*-overexpressing fruit during ripening (Fig. 5D). Altogether, these data suggest that MaMYB69 promotes banana fruit ripening and softening by stimulating the expression of genes involved in softening.

### MaMYB69 interacts with MaERF55 and itself to strengthen its regulation activity

**Figure 6:**
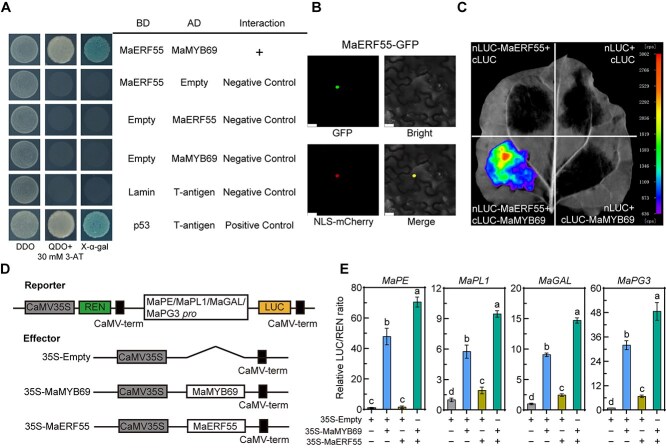
MaMYB69 interacts with MaERF55. (A) Y2H assay. The CDS of *MaMYB69* and *MaERF55* were cloned into pGADT7 and pGBKT7 vectors, respectively, and then introduced into the Y2H Gold yeast strain. A positive interaction was recorded if transformants grew well on selective medium (QDO) containing X-α-gal plus 30 mM 3-AT. (B) Subcellular localization analysis of MaERF55 in plant cells. NLS-mCherry was used as a nuclear marker. Bar = 20 μm. (C) LCI assay. *MaMYB69* and *MaERF55* were fused with the cLUC and nLUC, after which they were co-infiltrated into tobacco leaves. The combinations with empty vectors were used as negative controls. Luminescence signals were detected at 2 days post-agroinfiltration. (D) Schematic illustration of reporter and effector vectors. (E) Modulation of cell wall-modifying genes by MaMYB69 and MaERF55. Effector and reporter vectors were cotransfected into rice protoplasts to detect LUC and REN activities. The LUC/REN ratio for the empty and promoter vector combination is defined as 1. Symbols + or − denote the presence or absence of the effector in the combinations, respectively. Statistically significant differences (*P* < 0.05) identified by one-way ANOVA are indicated by distinct lowercase letters.

MYB TFs form complexes with other TFs, which modulate fruit ripening and quality [39]. We conducted a Y2H assay using a fruit cDNA library and revealed MaERF55 as a candidate interacting partner of MaMYB69. Expression profile analysis showed that *MaERF55* expression was significantly induced during banana fruit ripening (Fig. S5). The interaction between MaMYB69 and MaERF55 was verified by performing a Y2H assay. When AD-MaMYB69 and BD-MaERF55 were cointroduced into yeast Y2H Gold strain, the transformed cells grew well and turned blue in the QDO medium containing X-α-gal plus 30 mM 3-AT, indicating that MaMYB69 interacted with MaERF55 in yeast (Fig. 6A). This interaction was further confirmed *in vivo*. The subcellular localization of MaERF55 was studied by fusing MaERF55 with GFP protein and introducing it into the tobacco leaves. Strong GFP and mCherry signals were detected in the nucleus (Fig. 6B), confirming the nuclear localization of MaERF55 and suggesting a potential interaction with MaMYB69 in this compartment. In the luciferase complementation imaging (LCI) assay, *MaERF55* was inserted into pCAMBIA1300-nLUC to produce nLUC-MaERF55, whereas *MaMYB69* ligated to pCAMBIA1300-cLUC produced cLUC-MaMYB69. A strong fluorescent signal could only be observed when nLUC-MaERF55 and cLUC-MaMYB69 were coexpressed in tobacco leaves (Fig. 6C), further substantiating the interaction between MaMYB69 and MaERF55.

We performed DLR assays in rice protoplasts to determine whether MaERF55 affects the modulation of MaMYB69 to the target genes. As illustrated in Fig. 6D, the CDS of *MaMYB69* and *MaERF55* were separately introduced into the effector plasmid, whereas the *MaPE*, *MaPL1*, *MaGAL*, and *MaPG3* promoters were individually incorporated into the reporter plasmid. MaMYB69 significantly activated the promoter activities of *MaPE*, *MaPL1*, *MaGAL*, and *MaPG3* (Fig. 6E), consistent with the previous findings. MaERF55 enhanced the promoter activities of *MaPL1*, *MaGAL*, and *MaPG3*; however, it exerted no significant effect on the *MaPE* promoter (Fig. 6E). Notably, the coexpression of *MaMYB69* and *MaERF55* raised the transcriptional activity of these target genes compared with either factor expressed individually, indicating that their interaction strengthened the regulatory effect (Fig. 6E). In addition, MaMYB69 interacted with itself in yeast and plant cells (Fig. S6), indicating that it forms a homodimer for gene modulation.

### *MaMYB69* is directly activated by MabZIP5

**Figure 7:**
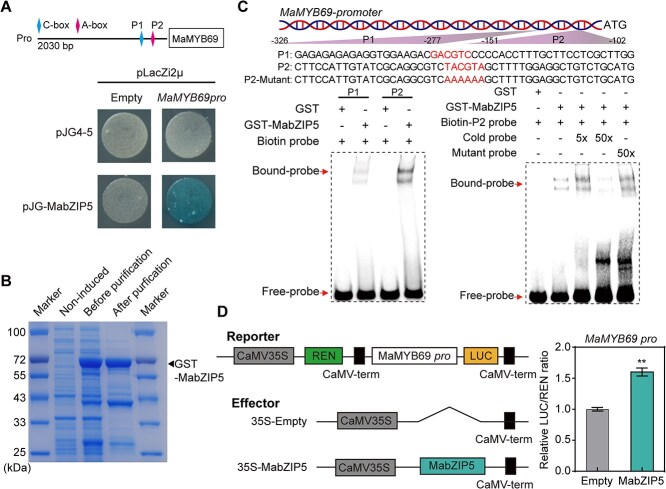
MabZIP5 activates *MaMYB69* expression by directly targeting its promoter. (A) Y1H assay showing the interaction of MabZIP5 with the *MaMYB69* promoter. The CDS of *MabZIP5* and the promoter of *MaMYB69* were cloned into the pJG4–5 and pLacZi2μ vectors, respectively, and co-introduced into EGY48 yeast cells. Transformed cells were grown on SC/−Trp/-Ura medium containing X-gal. Empty pJG4–5 vector and promoter fragment served as negative control. In the illustration of the genomic organization of the *MaMYB69* promoter, magenta diamonds indicate C-box and A-box motifs, while P1 and P2 represent two fragments on the MaMYB69 promoter. (B) Purified recombinant GST-MabZIP5 was analyzed by SDS-PAGE with Coomassie blue staining. (C) Interaction of MabZIP5 with the A-box in the *MaMYB69* promoter determined by EMSA assay. The label and mutant probes from the *MaMYB69* promoter are shown at the top. GST protein was used as a negative control. Competition experiments employed five-fold and 50-fold molar excesses of unlabeled probes, along with a 50-fold excess of mutant probes. Symbols + and – indicate presence or absence of protein/probe, respectively. (D) DLR assay showing that MabZIP5 promotes *MaMYB69* transcription in rice protoplasts. The left panel indicates the structures of reporter and effector vectors. The LUC/REN ratio for the empty and promoter vector combination is defined as 1. Double asterisks (**) mark significant differences at *P* < 0.01 (Student’s *t*-test).

To identify the upstream regulatory factors of *MaMYB69*, we performed a yeast one-hybrid (Y1H) screening assay against the cDNA library of banana fruit using its promoter as a bait. A fragment corresponding to the MabZIP5 was identified. Recently, MaZIP5 has been reported as a flavor activator, which directly enhances the expression of aroma biosynthetic genes [40]. To confirm this result, we conducted a Y1H assay using pJG-MabZIP5 and pLacZ-MaMYB69pro vectors and showed that yeast cells coexpressing MabZIP5 and MaMYB69pro grew well and turned blue on selective medium (Fig. 7A), indicating that MabZIP5 bound to the *MaMYB69* promoter. bZIP TFs favorably target DNA sequences with an ‘ACGT’ core sequence, such as G-box (CACGTG), C-box (GACGTC), and A-box (TACGTA) [41]. The *MaMYB69* promoter contained two ACGT core motifs (Text S1). An EMSA was conducted using biotin-labeled P1 (G-box) and P2 (A-box) probes to validate whether MabZIP5 can recognize the ACGT core motif in the *MaMYB69* promoter. Fusion protein GST-MabZIP5 with the P2 probe generated binding complexes, whereas a very faint band appeared with the P1 probe (Fig. 7B and C). Further EMSAs using the P2 probe showed that the shifted band was eliminated by the excess unlabeled P2 probe, whereas the mutant probe did not affect the binding signal (Fig. 7C). Considering the transcriptional activation activity of MabZIP5 [40], we hypothesized that MabZIP5 activates the transcription of *MaMYB69*. The DLR assay showed that coexpression of MabZIP5 with MaMYB69pro significantly enhanced the LUC/REN ratio versus empty vector control (Fig. 7D). These observations confirmed that MabZIP5 directly bound to *MaMYB69* and activated its expression.

## Discussion

Fruit softening, an essential ripening indicator, requires the participation of multiple enzymes and proteins that sequentially and coordinately modify the cell wall structure, ultimately determining the postharvest quality and shelf life of fruit [42]. Banana has multiple texture modification genes, such as PGs, PEs, PMEs, PLs, XETs/XTHs, β-Gal, and EXP, whose expression levels increase during fruit maturation [43, 44]. In the present work, the expression levels of *MaPE*, *MaPL1*, *MaGAL*, and *MaPG3* were significantly motivated by ethylene during banana fruit ripening (Fig. S5). This result agrees with previous reports [43, 44]. These data suggest that these genes are closely associated with fruit softening; however, the regulatory mechanisms governing their transcription remain unclear. Genetic engineering strategies to manipulate TF expression may be used to substantially delay softening, extending the shelf life and maintaining the quality of fruit. Thus, the identification of TFs that specifically modulate fruit softening has recently attracted attention in postharvest biology.

In plants, R2R3-MYB TFs have been linked to multiple physiological and biochemical activities, encompassing fruit ripening and softening. For example, in apple, MdMYB44 enhances fruit crispness by directly targeting the *MdMPE3* promoter and activating its transcription [31]. Two papaya MYB proteins, CpMYB1 and CpMYB2, function as transcriptional repressors, inhibiting cell wall degradation by directly suppressing the promoters of *CpPME1*, *CpPME2*, and *CpPG5* [45]. FvMYB79 activates the transcription level of *FvPME38*, hastening strawberry fruit softening [30]. In banana, 280 R2R3-MYB genes have been characterized [37], among which MaMYB3, MaMYB4, MaMYB44, MaMYB62 exhibits decreased expression during fruit ripening, and have been associated with fruit maturation by governing ethylene production, starch metabolism, and texture modification [28, 32, 44]. Recent report indicates that an R1-MYB gene, MaMYB16, participates in fruit ripening by repressing starch degradation through its active isoform, MaMYB16L [46]. Moreover, we found that all these examined MYB TFs serve as transcriptional repressors during fruit ripening, implying their significant importance in managing ripening initiation. In this research, we examined the expression levels of all upregulated-MYB TFs using our transcriptome data. The expression of MaMYB69, MaMYB151, and MaMYB177, which contained the typical conserved R2R3-type domain and were localized to the nucleus, showed significant transcriptional activation and reached high transcript abundance during banana ripening (Figs 1, 2A and Figs S1, S3, and  S4A), suggesting that they modulate banana ripening. MaMYB69 showed high homology with AtMYB107 and AtMYB9 in *Arabidopsis*, whereas MaMYB151 and MaMYB177 clustered with AtMYB56 (Fig. S2). Our results suggest that MaMYB69 possesses transcriptional activation activity, with its activation domain located at the C-terminus (326–348 aa) (Fig. 2B–D), functioning as a transcriptional activator and indicating that MaMYB69 is a novel positive modulator of fruit ripening. Although, MaMYB151 and MaMYB177 are localized in the nucleus, they do not exhibit transactivation activity (Fig. S4), suggesting that they may require other transcriptional activators to cooperatively govern the expression of their target genes. Moreover, a study reported that their closest homolog AtMYB56 plays a role in modulating sugar and anthocyanin accumulations in *Arabidopsis,* [47], thus speculating that MaMYB151 and MaMYB177 influence the sugar and pigment metabolism during banana fruit ripening.

MYB TFs exert their regulatory roles through specific recognition and interaction with *cis*-acting elements (e.g. AC-box, MBS, and MYBCORE) in target gene promoters [48]. A recent study has demonstrated that EjMYB1 in loquat directly stimulates the expression of lignin synthesis genes by binding to the AC-box in the promoter, thereby contributing to flesh lignification during chilling injury, and EjMYB2 counters EjMYB1-mediated induction by competitively binding to the AC-box [49]. Moreover, PbrMYB24 binds the AC-box in the *Pbr4CL1* and *PbrCCOAOMT1* promoters and the MBS in the *PbrCESA8b* promoter to control lignin and cellulose production in pear stone cells [48]. Recently, tomato SlMYB75 has been shown to promote aroma volatile and anthocyanin accumulation by stimulating the transcription of *AADC2*, *LOXC*, and *TPS* with AC-boxes-included promoters [26]. In the present study, MaMYB69 bound to the AC-box in the *MaPE*, *MaPL1*, *MaGAL*, and *MaPG3* promoters, activating their expression (Fig. 3). *MaMYB69* overexpression in tomato promotes fruit ripening by increasing the transcription of *SlPL*, *SlPE3*, *SlTBG4*, and *SlPG2* and fastening ethylene production, fruit softening, and color change (Fig. 4). Promoters of these tomato target genes containing multiple AC-boxes, suggesting that MaMYB69 directly binds to these *cis*-elements and activates their expression in tomato. Consistently, transient overexpression of *MaMYB69* in banana fruit accelerated fruit ripening and softening by enhancing the expression of *MaPE*, *MaPL1*, *MaGAL*, and *MaPG3* (Fig. 5). These results reinforce that MaMYB69 positively controls fruit ripening. Notably, in addition to the alteration observed in fruit firmness, *MaMYB69* overexpression in tomato and banana exhibited alterations in ethylene production (Figs 4 and 5). DLR and EMSA experiments demonstrated that MaMYB69 directly interacted with the *MaACO1* promoter to enhance its expression (Fig. S7), which corresponded to a change in ethylene production. The results indicate that MaMYB69 can directly interact with the *MaACO1* promoter to enhance its expression (Fig. S7), but MaMYB69 shows a relatively weaker transcriptional activation to *MaACO1* than cell wall degrading-related genes (Fig. 3B and Fig. S7A). Collectively, these findings suggest that MaMYB69 participates in fruit ripening by predominantly affecting the cell wall degradation pathway. In addition, MaMYB69 interacted with itself and self-activated its own promoter (Fig. 3 and Fig. S6), indicating its feedback regulatory manner. Similarly, MaWRKY49, MaWRKY111, MabZIP21, and MaNAC169 interact with themselves to form a dimer and enhance their own transcription [35, 50, 51], indicating a potential mechanism for amplifying the modulation activity and function of TFs.

Previous research has established that MYB TFs modulate target gene expression through interactions with co-regulators and molecular chaperones. A well-characterized example is the MYB–bHLH–WD40 complex, which serves as a key modulator in controlling anthocyanin biosynthesis in plants [24]. In banana, two R2R3 MYB activators, MaMYBPA1 and MaMYBPA2, interact with MaMYC (bHLH TF) and MaTTG1 (a WD40 protein) to form functional complexes that transactivate *MaANS*, *MaANR*, and *MaLAR* promoters, promoting anthocyanin biosynthesis [52]. In pear, PcERF5 enhances anthocyanin synthesis and accumulation by forming a dimer with PcMYB10 [39]. In loquat, EjAP2–1 controls flesh lignification in response to chilling stress by interacting with EjMYB1 and EjMYB2 [53]. In strawberry, the interaction between FaERF#9 and FaMYB98 reinforces the FaMYB98-mediated transcriptional activation capacity of *FaQR*, contributing to the biosynthesis of aroma in strawberry fruit [54]. In this study, we determined a novel AP2/ERF family TF, MaERF55, which interacted with MaMYB69 (Fig. 6A–C). This result is similar to that observed in previously mentioned studies and further suggests that the MYB–ERF complex is ubiquitous in plants. MaERF55 significantly increased MaMYB69 activation for the expression of *MaPE*, *MaPL1*, *MaGAL*, and *MaPG3* by forming a protein dimer (Fig. 6E). We additionally found that MaERF55 individually activated the promoters of *MaPL1*, *MaGAL*, and *MaPG3* (Fig. 6E). Furthermore, results from the Y1H assay demonstrated that MaERF55 binds directly to the *MaGAL* and *MaPG3* promoters, but not to the *MaPL1* promoter (Fig. S8). These findings suggest that MaERF55 is involved in cell wall degradation through direct activation of *MaGAL* and *MaPG3* and indirect control of *MaPL1*.

Fruit ripening is governed by intricate networks featuring multistep transcriptional cascades orchestrated by TFs. Anthocyanin synthesis in fruits is adjusted by various bZIP TFs that target MYB TFs. For instance, in apple, the anthocyanin activator MdMYB114 acts downstream of MdbZIP4-like [55]. Similarly, in pear, bZIP TF PyHY5 stimulate the promoter of *PyMYB10* to promote anthocyanin biosynthesis [56]. Moreover, bZIP TFs play vital roles in banana fruit ripening and quality formation by modulating gene expression. MabZIP93 and MabZIP21 facilitate fruit softening via direct binding and activation of genes encoding cell wall degradation enzymes [50, 57]. In this study, a bZIP TF and transcriptional activator, MabZIP5 [40], whose expression is activated during banana ripening (Fig. S5), directly targeted the *MaMYB69* promoter, inducing its transcription (Fig. 7). Therefore, we can reasonably assume that MabZIP5 activates *MaMYB69* transcription, which elevates the MaMYB69-mediated transactivation of cell wall-modifying genes *MaPE*, *MaPL1*, *MaGAL*, and *MaPG3*, ultimately accelerating fruit softening and ripening. In addition, we found that the promoters of *MaPE*, *MaPL1*, and *MaGAL* each contain one MabZIP5-binding motif. DLR and EMSA assays revealed that MabZIP5 can directly target the *MaPL1* promoter to activate its transcription, with no effect observed on *MaPE* and *MaGAL* (Fig. S9), indicating that MabZIP5 may be directly involved in cell wall degradation by enhancing *MaPL1* transcription. Moreover, MabZIP5 acts as an aroma formation activator during banana ripening [40]. Our results further enhance our knowledge of the regulation mechanism of MYBs and bZIPs and enrich the complex molecular network of fruit maturation in banana.

In summary, we identified that an R2R3-type MYB TF, *MaMYB69*, is induced by ethylene and governed by MabZIP5 activation. Moreover, MaMYB69 directly targets the promoters of *MaPE*, *MaPL1*, *MaGAL*, *MaPG3*, and itself, activating their expression. In addition, MaMYB69 recruits MaERF55 to enhance its transcriptional influence of target genes, promoting cell wall degradation during banana ripening. This study elucidates the molecular mechanisms underlying cell wall degradation mediated by MaMYB69, advancing our comprehension of the regulatory cascade governing fruit softening in banana.

## Materials and methods

### Banana fruit and treatment

Baxijiao (*Musa acuminata*, AAA group) fruit at the three-quarter stage was harvested from an orchard in Guangzhou, China. The fruit was divided into individual fingers and immersed in fungicide (0.05% sporgon) for 5 min to reduce the risk of disease. Fruit samples were processed according to a previous report [20]. In brief, fruit fingers were categorized into three treatment groups: natural ripening (no treatment), ethylene-induced ripening (treated with an 800 ppm ethephon aqueous solution for 1 min), and 1-MCP-delayed ripening (exposed to 1 mg/l 1-MCP for 22 h). The treated fruit was subsequently maintained in a constant temperature and humidity chamber (22°C, 85%–90% relative humidity) until full maturity. Pulp tissue was collected at different intervals and preserved at −80°C for further use. Each treatment was performed in three biological replicates.

### Molecular characterization and gene transcription profile analysis

The online tool ExPASy (https://web.expasy.org/) and WoLF PSORT software (https://wolfpsort.hgc.jp/) were used to analyze the MW and subcellular localization of the proteins, respectively. The MUSCLE program was employed to align protein sequences of MaMYB69, MaMYB151, and MaMYB177 with *Arabidopsis thaliana* MYB members, followed by phylogenetic tree construction via the neighbor-joining method in MEGA X software. Multiple sequence alignment were visualized and analyzed using Jalview 2.11.3.0 software. Total RNA extraction, cDNA synthesis, and gene expression analysis were performed following previously described protocols [58]. Target gene expression levels were calculated relative to the *MaCAC* reference gene (HQ853240) through the 2^−∆∆Ct^ method, and all primer sequences are provided in Table S1.

### Subcellular localization and transcriptional activation analysis

To construct GFP fusion proteins, we cloned the coding sequences (excluding stop codons) of MaMYB69, MaMYB151, MaMYB177, and MaERF55 into the pEAQ-HT-GFP vector, yielding MaMYB69-GFP, MaMYB151-GFP, MaMYB177-GFP, and MaERF55-GFP, respectively. These recombinant plasmids along with the pEAQ-GFP control were transformed into *Agrobacterium tumefaciens* GV3101 and subsequently infiltrated into leaves of 4-week-old *Nicotiana benthamiana* plants. Fluorescence signals were examined 48 h postinfiltration using a Zeiss LSM710 laser scanning confocal microscope (Zeiss, Oberkochen, Germany).

For transactivation analysis of MaMYB69, MaMYB151, and MaMYB177, the CDS of *MaMYB69*, *MaMYB151*, and *MaMYB177* were cloned into pGBKT7 vectors and subsequently introduced separately into yeast cells (Y2H Gold). Transformed cells were spotted on SD/−Trp medium, incubated for 3 days at 30°C, and selectively grown on SD/−Trp/-His/−Ade medium with or without X-α-gal to determine the transactivation activity. Moreover, the truncated fragments containing different protein domains were amplified using PCR to identify the transcriptional activation region of MaMYB69. They were additionally inserted into pGBKT7 vectors and introduced into Y2H Gold. Yeast transformation was performed as described for Matchmaker™ GoldYeast Two-Hybrid Systems (TaKaRa, Dalian, China).

### Dual-luciferase reporter assays

To examine the transactivation capability of MaMYB69, MaMYB151, and MaMYB177, we constructed the pBD-62SK-MaMYB69, pBD-62SK-MaMYB151, and pBD-62SK-MaMYB177 vectors as effectors and the pGreenII 0800–5 × GAL4-TATA-LUC plasmid as the corresponding reporter. The CDS of *MaMYB69*, *MaERF55*, and *MabZIP5* were ligated into the pGreenII 62-SK plasmid to generate effector vectors. Approximately 2 kb of the upstream sequences of the initiation codon of *MaPE*, *MaPL1*, *MaGAL MaPG3*, and *MaMYB69* were cloned into pGreenII 0800-LUC plasmid as reporter constructs to determine the influence of TFs to their target genes. The empty pGreenII 62-SK vector was considered as a negative control. The effector and reporter plasmids were co-delivered into rice protoplasts using the PEG-CaCl_2_-mediated transformation approach. After 18 h of transformation, the LUC activity was measured using a DLR assay system (Promega, WI, USA).

### Electrophoretic mobility shift assay

The entire *MaMYB69* and *MabZIP5* CDS were subcloned into the pGEX-4 T-1 plasmid to generate GST-MaMYB69 and GST-MabZIP5, respectively. The recombined constructs were introduced into chemically competent cells of *E. coli* strain Rosetta (DE3) (Beyotime, Nantong, China) to express GST-fusion proteins. We conducted protein expression at 28°C for 6 h by adding 1 mM isopropyl β-D-thiogalactopyranoside to the cell culture, and GSTSep Glutathione Agarose Resin 4FF (Yeasen, Shanghai, China) was used to purify the recombinant proteins following the manufacturer’s protocol. The synthesis and modification of the probes were performed by Sangon Biotechnology (Shanghai, China). Unlabeled probes were used as cold probes to compete with labeled probes. The predicted binding site in the mutant probe was modified to the AAAAAA fragment to verify the TF binding specificity. An EMSA was conducted using a Chemiluminescent EMSA Kit (Beyotime, Nantong, China) following previously established methods [35]. All primers used for EMSA are provided in Table S1.

### Yeast two-hybrid and firefly luciferase complementation imaging assay

The CDS of *MaMYB69* was introduced into the pGADT7 plasmid as prey, and the *MaMYB69-F* fragment and coding region of *MaERF55* were inserted into the pGBKT7 plasmid as bait. The prey and bait plasmids were cotransfected into Y2H Gold yeast cells. The cultures were incubated in synthetic dropout nutrient medium (SD/−Trp/−Leu) (DDO) at 30°C for 3 days and subsequently spotted on synthetic dropout nutrient medium (SD/−Trp/−Leu/-His/−Ade) (QDO) with X-α-gal for potential interactions assessment.

Full-length *MaMYB69* was fused to the pCAMBIA1300-nLuc and pCAMBIA1300-cLuc plasmids, and the CDS of *MaERF55* was inserted into pCAMBIA1300-nLuc plasmid. The fusion constructs were introduced into GV3101 strain and infiltrated into tobacco (*N. benthamiana*) leaves. nLUC-MaERF55 and nLUC-MaMYB69 were mixed with cLUC-MaMYB69 in equal proportions. The mixture of empty and fusion vectors was used as the negative control. After agroinfiltration for 2 days, the leaves were injected with 1 mmol/l D-luciferin. Fluorescence images were captured and processed using the NightSHADE LB985 plant imaging system (Berthold, Bad Wildbad, Germany).

### Yeast one-hybrid assay

The Y1H assay was conducted as previously described [35]. In brief, the 2-kb promoter sequence of *MaMYB69* was cloned into the reporter vector pLacZi2μ, and the CDS of *MabZIP5* was inserted into the pJG4–5 vector. The pLacZ-MaMYB69pro plasmid was cotransformed with the pJG-MabZIP5 plasmid into competent cells of yeast EGY48. The empty pJG4–5 and pLacZ-MaMYB69pro vectors were used as the negative control. Transformed cells were grown on SD/−Trp/-Ura medium at 30°C for 2 days. Subsequently, single colonies were used to inoculate SC/−Trp/-Ura selection medium including raffinose (Raf), galactose (Gal), BU buffer, and X-gal for further screening.

### Tomato transformation analysis

The CDS of *MaMYB69* was cloned into the pBWA(V)HS-Flag plasmid to create an overexpression construct. The recombinant construct was subsequently introduced into *Agrobacterium* GV3101 and transformed into Micro-Tom (*Solanum lycopersicum*, ‘Micro-Tom’) [59]. Transgenic plants were screened on the selective medium containing hygromycin. The positive transformants were further confirmed using qRT-PCR. The transgenic tomato plants were cultivated in a plant culture room at 24°C under a 16-h light/8-h dark cycle, with 60% relative humidity and 200 μmol m^−2^ s^−1^ light intensity. Three *MaMYB69-*overexpressing lines (T2 generation) were selected for further experiments. Tomato fruits were collected at 26, 32, 35, and 38 DPA for the determination of fruit firmness, color index, and ethylene production as described previously [35].

### Transient overexpression analysis in banana fruit

The CDS of *MaMYB69* was inserted into the pCXUN-HA vector containing a ubiquitin promoter (Ubi) to investigate the role of MaMYB69 in banana fruit ripening. The recombinant plasmid and empty plasmid were individually transformed into *A. tumefaciens* strain EHA105, following which they were injected into the tail of mature green banana fruits in accordance with methods described previously [35]. Banana fruits were incubated at 22°C for 1 day after infiltration, treated with 100 ppm of ethylene for 16 h, and ripened at 22°C. Samples were collected at Days 0, 1, 2, 3, 4, and 5 to assess physiological parameters such as firmness, peel color, and ethylene production.

### Gene accessions

Sequence data used in this study are available from the Banana Genome Hub and the Solanaceae Genomics Network under the following accession numbers: *MaMYB69* (Ma04_g16770), *MaERF55* (Ma06_g11920), *MabZIP5* (Ma05_g26820), *MaMYB151* (Ma06_g33920), *MaMYB177* (Ma07_g23230), *MaPE* (Ma03_g05660), *MaPL1* (Ma06_g30000), *MaGAL* (Ma07_g08800), *MaPG3* (Ma02_g04450), *MaACS1* (Ma04_g35640), *MaACO1* (Ma07_g19730), *SlPE3* (Solyc07g064180), *SlPL* (Solyc03g111690), *SlTBG4* (Solyc12g008840), and *SlPG2* (Solyc10g080210).

### Statistical analyses

The data are presented as mean ± standard deviation (SD). Statistical analyses were conducted using SPSS 25.0 (IBM, Armonk, NY, USA), and diagrams were generated using GraphPad Prism 8 (GraphPad Software, CA, USA). Between-group differences were assessed through Student’s *t*-test for pairwise comparisons, whereas one-way ANOVA followed by Duncan’s test was applied for comparisons among multiple groups.

## Supplementary Material

Web_Material_uhaf275
