# Supplementary material for: The MabZIP5–MaMYB69 module cooperates with MaERF55 to modulate banana fruit ripening via cell wall degradation
Source: Hortic Res. 2025 Oct 17;13(1):uhaf275. doi: 10.1093/hr/uhaf275 (PMC12881857; doi:10.1093/hr/uhaf275)
Supplement: Web_Material_uhaf275 [file web_material_uhaf275.zip › Figure S1-S9-09.16.docx]

**
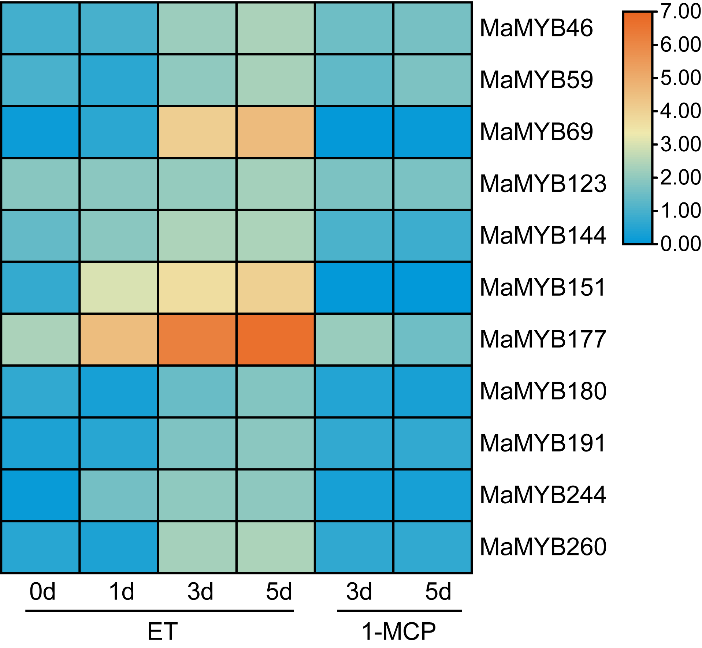
**

**Figure S1.** Expression profile visualized as heatmaps of upregulated *MaMYB* genes in RNA-seq data during banana fruit ripening.

**
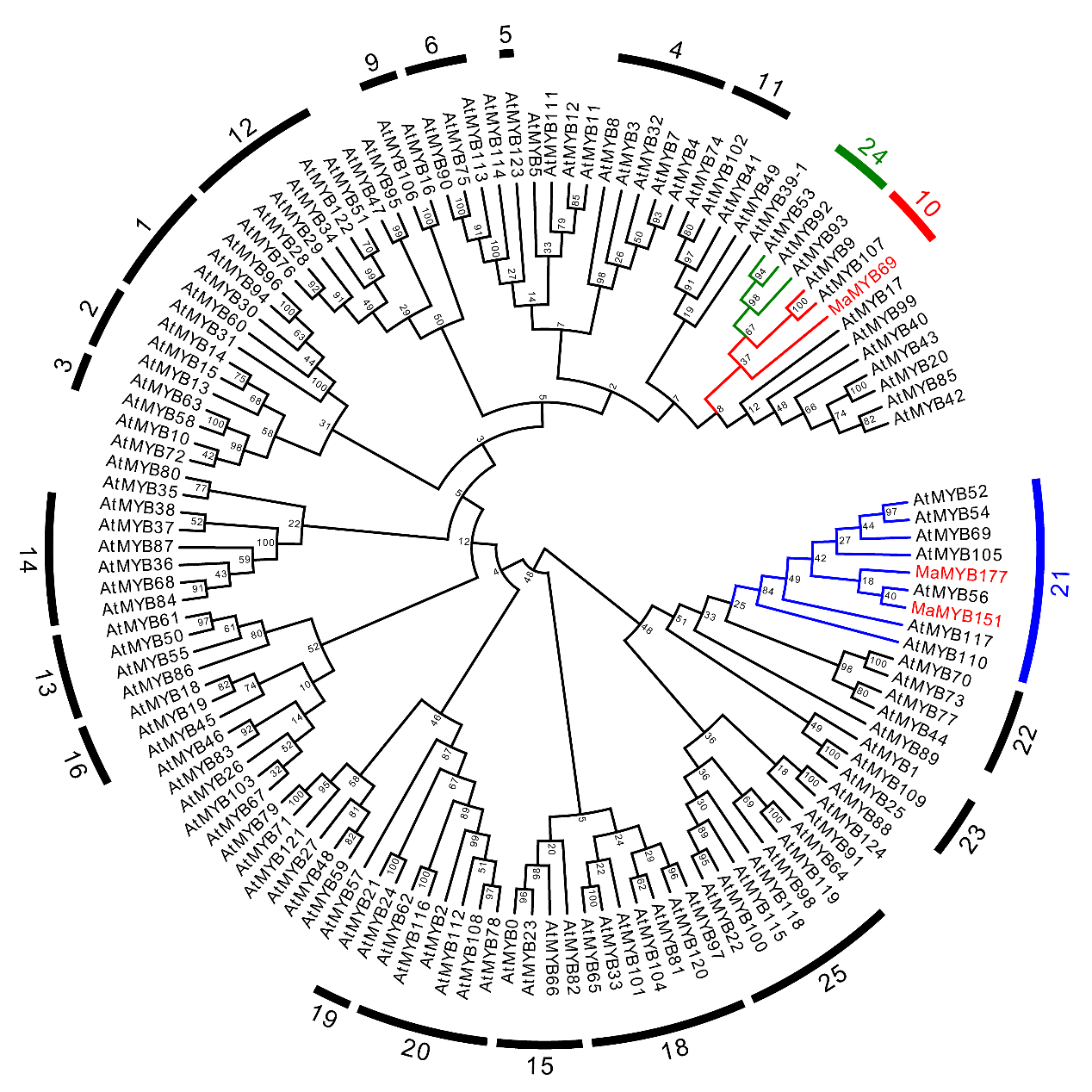
**

**Figure S2.** Phylogenetic analysis of MaMYB69/151/177 with *Arabidopsis* MYB proteins. MaMYB69 is marked in red. Phylogenetic analysis was performed with MEGA X, applying the neighbor-joining approach.

**
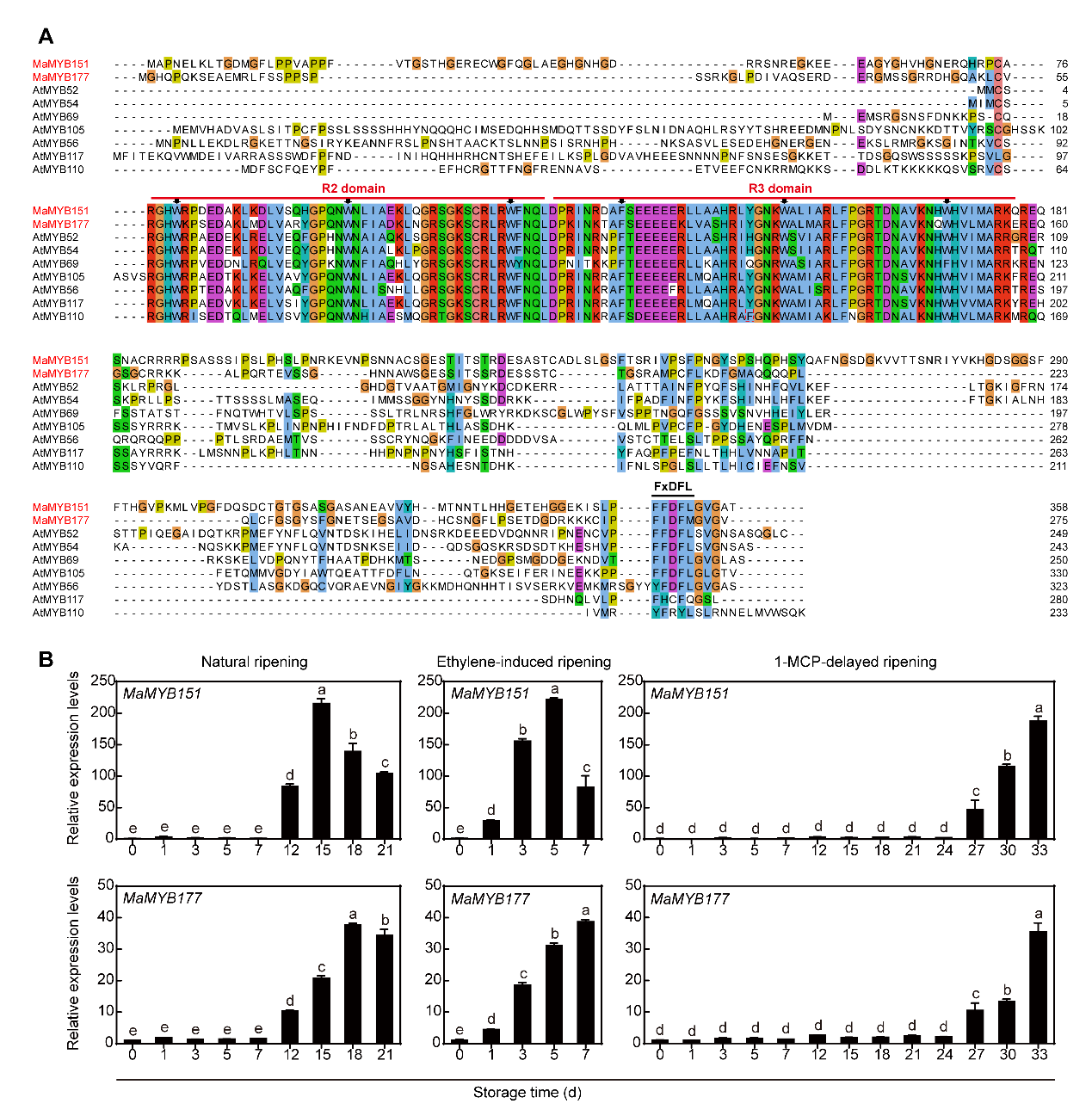
**

**Figure S3.** Sequence analysis and expression pattern of MaMYB151 and MaMYB177. (**A**) Alignment of amino acid sequence of of MaMYB151 and MaMYB177 with *Arabidopsis* MYBs. Red lines represent R2 and R3 MYB conserved domains. The black arrows denote the conserved amino acids tryptophan (W) and phenylalanine (F). The FxDFL motif in the C-terminal is denoted by a black line. (**B**) Expression patterns of *MaMYB151* and *MaMYB177* in pulp tissue. Results are presented as mean ± standard deviation (SD) from three independent replicates. Statistically significant differences (*P* < 0.05) identified by one-way ANOVA are indicated by distinct lowercase letters.


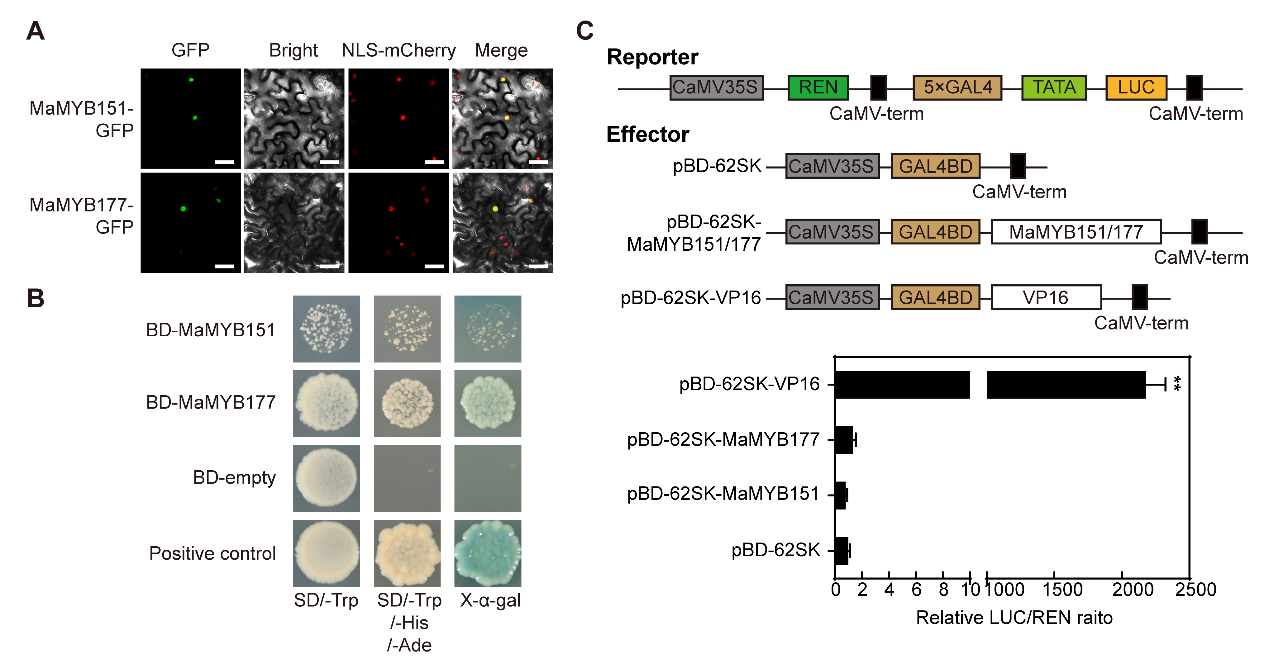


**Figure S4.** Molecular characterization of MaMYB151 and MaMYB177. (**A**) Subcellular localization of MaMYB151 and MaMYB177 in tobacco leaves. Transient expression of GFP alone served as a control. Bar = 40 μm. (**B**) Identification of transactivation capacity of MaMYB151 and MaMYB177 in yeast cells. The pGBKT7-p53/pGADT7-T-antigen pair served as a positive control and the pGBKT7 (BD-empty) was employed as a negative control. (**C**) Transcriptional activity analysis of MaMYB151 and MaMYB177. Upper panel shows the schematic diagram of the reporter and effector constructs. pBD-62SK and pBD-62SK-VP16 were employed as the negative control and positive control, respectively. The LUC/REN ratio for empty pBD-62SK and reporter vector combination was defined as 1. Symbol (**) marks significant differences at *P* < 0.01 (Student's *t*-test).

**
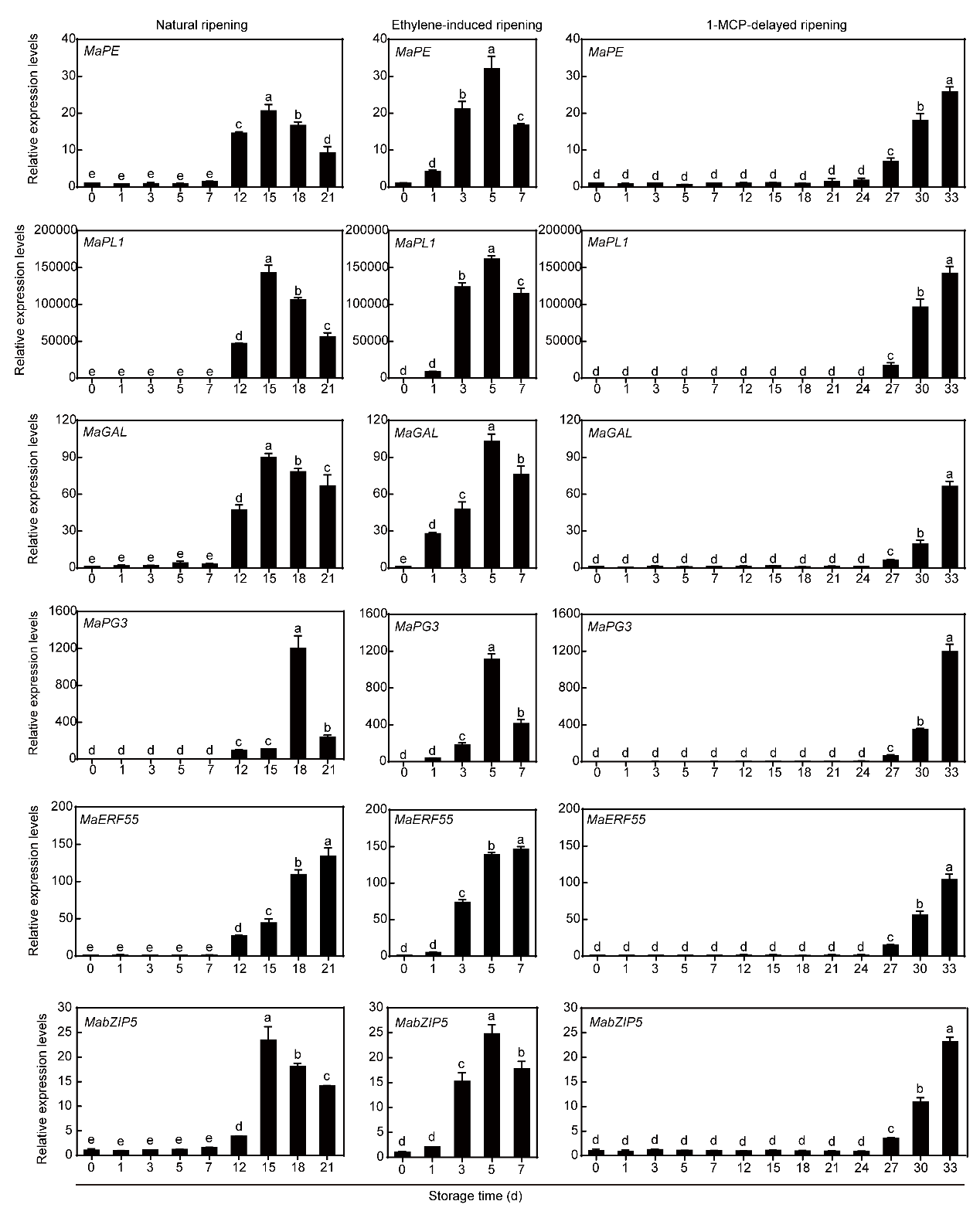
**

**Figure S5.** Transcription analysis of *MaPE*, *MaPL1*, *MaGAL*, *MaPG3*, *MaERF55*, and *MabZIP5* in banana pulp from different ripening treatments. Gene expression level of 0 d was defined as 1. Results are presented as mean ± standard deviation (SD) from three independent replicates. Statistically significant differences (*P* < 0.05) identified by one-way ANOVA are indicated by distinct lowercase letters.

**
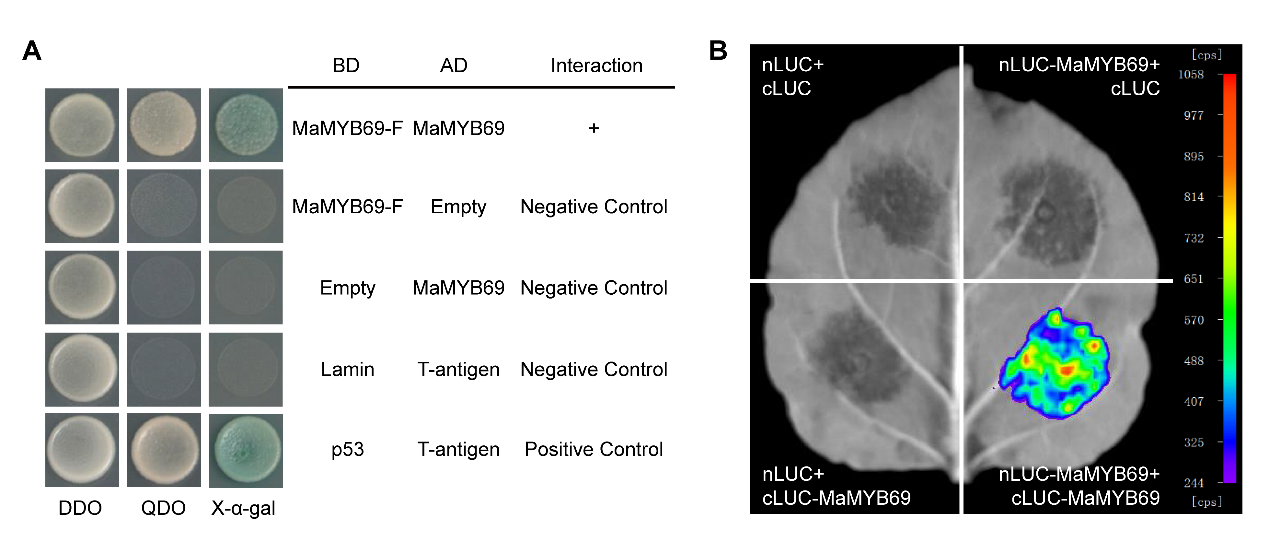
**

**Figure S6.** MaMYB69 interacts with itself. (**A**) Y2H assay. BD-MaMYB69-F and AD-MaMYB69 constructs were co-expressed in yeast. BD-p53 / AD-T-antigen pair and BD-Lamin / AD-T-antigen pair served as the positive control and negative control, respectively. DDO and QDO denoted the synthetic dropout nutrient medium (SD/-Trp/-Leu) and synthetic dropout nutrient medium (SD/-Trp/-Leu/-His/-Ade), respectively. (**B**) LCI assay. MaMYB69 was separately fused to nLUC and cLUC and co-expressed in tobacco leaves. MaMYB69 expression alone was used as a negative control.

**
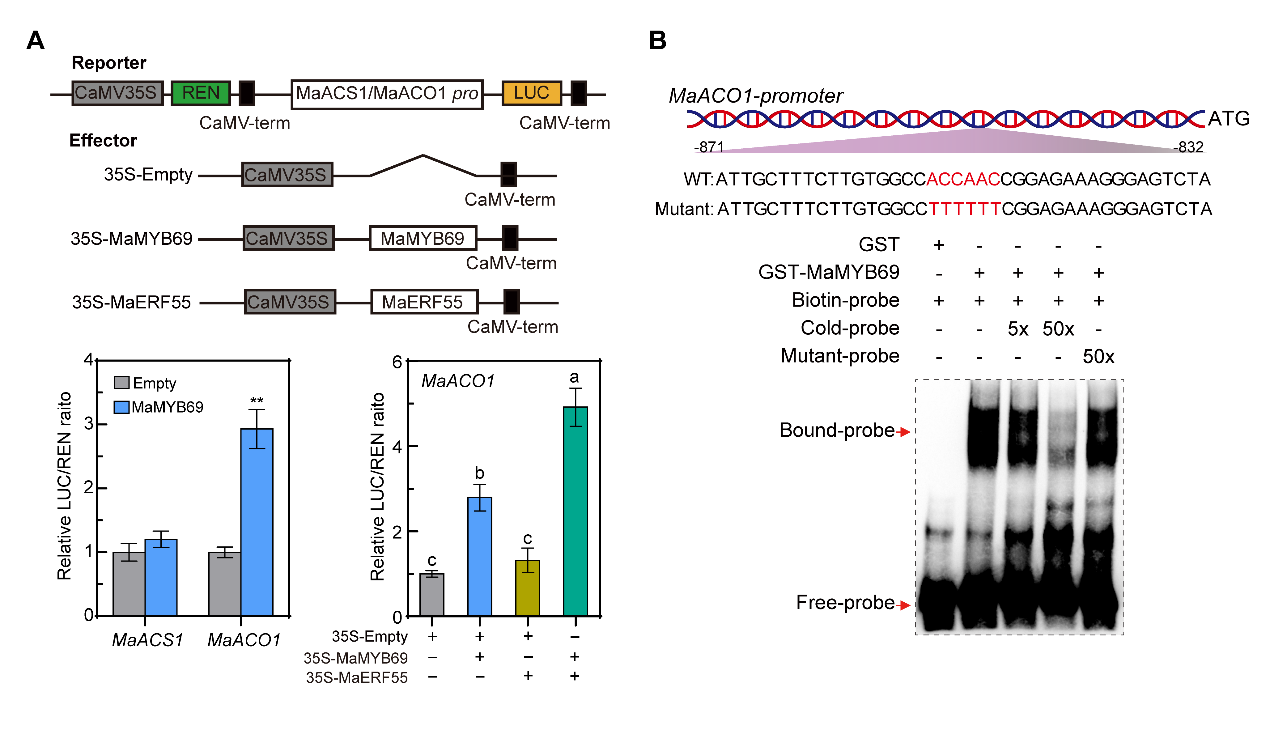
**

**Figure S7.** MaMYB69 cooperated with MaERF55 to augment the expression of *MaACO1*. (**A**) The cooperative interplay between MaMYB69 and MaERF55 significantly upregulated the expression levels of *MaACO1*. The structures of reporter and effector vectors are shown at the top of panel. Double asterisks and lowercase letters denote statistical significance at *P* < 0.01 (Student’s *t*-test) and *P* < 0.05 (one-way ANOVA), respectively. (**B**) EMSA assay demonstrated that MaMYB69 directly binds to the promoter regions of *MaACO1*. The labeled and mutant probes derived from the *MaACO1* promoter are displayed at the top, with GST protein serving as the negative control. The MaMYB69-binding motifs are marked by red color. +, presence; –, absence.


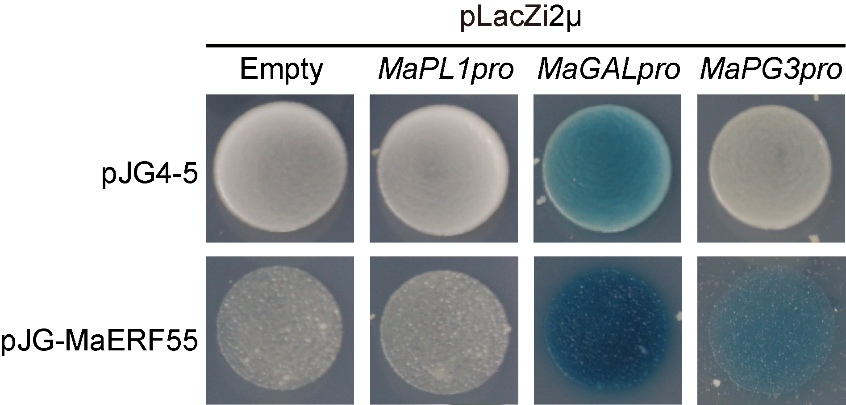


**Figure S8.** Y1H assays showed the interaction between MaERF55 and the promoters of *MaPL1*, *MaGAL,* and *MaPG3*. The binding ability was analyzed by a color change on SC/-Trp/-Ura medium following the addition of X-gal. The empty pJG4-5 vector and promoter fragment served as a negative control.


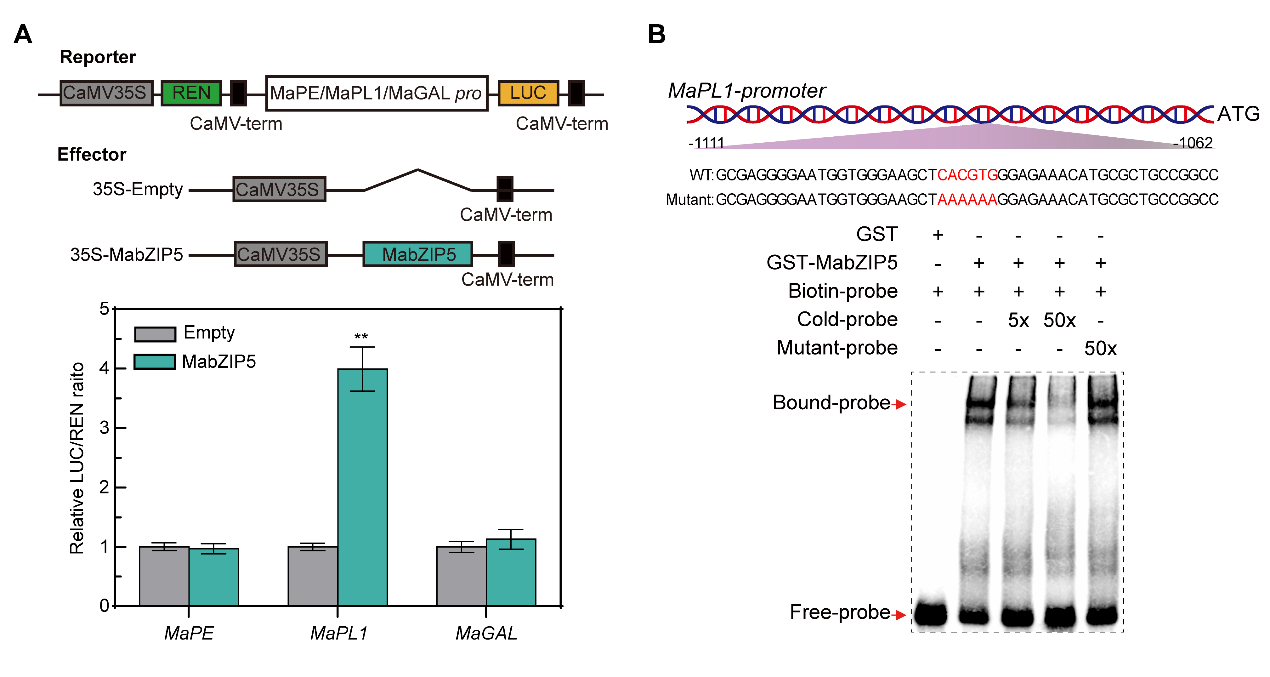


**Figure S9.** MabZIP5 directly targets the *MaPL1* promoter to activate its transcription. (**A**) Analysis of transcriptional regulation of *MaPE*, *MaPL1*, and *MaGAL* by MabZIP5. The reporter and vector structures are presented at the upper section of image. The LUC/REN ratio is normalized to 1 for the empty vector and promoter vector. Significant differences (*P* < 0.01, Student’s *t*-test) are marked with double asterisks. (**B**) EMSA assay showed that MabZIP5 directly targets the G-box motifs in the MaPL1 promoter. The probe sequences are depicted on the top of the image, where the MabZIP5-binding site is indicated by red letters. + and - are used to represent the presence and absence of the probe or protein, respectively.
