# Supplementary material for: The MabZIP5–MaMYB69 module cooperates with MaERF55 to modulate banana fruit ripening via cell wall degradation
Source: Hortic Res. 2025 Oct 17;13(1):uhaf275. doi: 10.1093/hr/uhaf275 (PMC12881857; doi:10.1093/hr/uhaf275)
Supplement: Web_Material_uhaf275 [file web_material_uhaf275.zip › Text S1-MaMYB69-0910.docx]

**Supplemental Text S1** Promoter nucleotide sequences of *MaPE*, *MaPL1*, *MaGAL*, *MaPG3*, *MaMYB69*, *MaACO1* and *MaACS1*. Red and yellow boxes represent MaMYB69 and MabZIP5 potential binding sites, respectively. Translation start site (ATG) was shown in green box.

**>MaPE (Ma03_g05660) promoter**

AGGAGGGACCATCAGAATCTAGATTCATAATACAAGAAATTTGTAATATTTTTTGGTTGTTAAGATTCATTGTGCGTCTCTATTACAACAAAATTTTTCTGGCTTTAGTTCTGTTGTACAATCGTCTCACCAAAGTCTCTATTGCATTCTATGCTTATGGTGGTTTCACTTAATGCCAATTCGATCTGAGTTTGTATTACTTCTTGTCAATACAAGAGAAAATAAATAGGTTATGAAGGCCCTATTGCATTTTCAATAAAAAAAATTAAGATTCCTGTTGGAGAACTTTTAGAGAACAAGATACTTCAGAATGTTGGCAGGATAGACCTAAAATCCGCATGTTCCAAGGATTTGGCTTATTTCTGATTGATCTGACTGATATAACCTAGCTGGTTGGGTTTTGACTGTTTTATAGGCTGGCGAGTTGTTGAGGTTCCGATTCTTGCATATCTTGCTGATTCCAAATGTTTTCCAGGAGATACTGCCAATCCAATTGCTCCCAACAGATTTTCAGTCACTTAGAAAGTTGTTTTGATTTCATGATTATACTCATCTGAATAAGTTGCTTATGCCAATATAGAAGAACCTGCAAGTTCAACTGCTTAACTTTTTCTGCAAATAATATTGTGTGATGTGTTTTATCGTTCATGGTTTCTATAGTAACTCGATTTCAACATGTCACTGGTCATTTTTAGCACTTATTTACGTATTGTCCTCTGAACTGATATCTACTTCCTTTCTAAAGATTTTGCAGCCCTCCAAGGATCTCGAGAGACTTCTTAACGGGGAGGGTATCAAGGACAGCATTAGTTGTACCACCATCTCTAGTATTTGCAAAACGTATGATCTTGACAGGAATGTTATAGTGAAGTACAGGAATTTACGATAGTTTCTTGAATTGCTTGGTGCATTCAGTGCTAATTACAAGTTTGAACTTGTTTTTACCGAGCTCACAACATGCAAACATCTTTTGCACAGCCTATGCATGCGACAAATGAGCTTAAGGCCAAAGTTTTGTTTACGGTAAATGTTTCATTGTTATAAAGTTAAATTGGACGAGGAATTTTTCTTTGACTCCTATATTGAATATGACAACTGAGTATTGATTGAGTGGAATGTTCTTCCTTTCTAATTGAAGTATCAATTCAAGTTTTTCAGCTTCTCCAAAATTTGTAATTGACTTCATGTTCTTTTGATAATTGATGTGAGATGACTATGCTACTCTTGGTGTAATAACAGGCAAGGTGGTTCATTTGGAGAGGCTTTGTGAATCCAATGTGTTGTAATCTGTTTGGTAATTCTAAACTTGTGCATCTATAAAGTATTCTAAACTTTTGCCCATATTTAAGATTTACATATTGTTCATTGAGGCCTTTCTAGATTTTAAAAGTGTTTTGGTTGATTATTGGTAGGAACATTTAAATCCTGATATGTTGATTTCCACTAACTGCATATTGTTTTTGAGTACCTAAATTGTTCTATGATTTTGAGGCGTGCTAACATCATCAAGGCTGAAGCATGAATCTGATGGTTCCTTGTGTCGATAGCTAAGAGATGGGGCGCCATAAGTAATCCACGTTTGATCTCTCTTCATTTCTAATCTTGTTTATTTCAGAATTTGCCCGACTGGTTAGTCAGTTTTGGTAGAATTAGCATGTGATATGATGTGTAATAGTGAAATAAGAGACGGAATTACGTGACAATGATGATAAAGAATGAAGACCTGAGAGGAAGAACGAGAAAAGGATGATGAAATATGTGTGTCGGTCTTTTGGATTTAGTGCAAACATCCGTCCATCTCATGCTACATTTGTCTTGTGATAGTCCTGTGATGAAAATATCTTCCTATTAAGGTGTCGATGGTGAAGTGTTTGGAGAAGTAGGAGCGGCAGCTCCACTGATATATTATCTCTTCCCATCTCTCTTCCTCCACCCTATTTAGCATCGGTGTCGTCCAACGCTACGTGACCTGACCCGAAAGCAGCATG

**>MaPL1 (Ma06_g30000) promoter**

CAAGTTGTAGTGCATGATAAAGAAAGATGTGCAGGTGTTAACCTTGGTGTTTGATGGAACACAAGACTCTTTATCAACAATATGATGACATTAGAACAAGCAGCTGACATGCATTACTTTTGATCAAGATAGGCTGCTCTTGATCCTCTCAGCAGCAGACAGAATGTACTTATTCTTGCTATTTGTCTCAATCATGCAGAATGATATGGCTAAATCAATACTTTCATTGAACAACTAGAACATGATTTCTCATAAATTTATCGGAAGATATATAATTAGACTCAAAACCAGCAGAACACTACACCAGTTGTTAGTTGCAGCAAAAAATAATATTGTCAACAAGAAGCTAGCAGCTATTTACTTCTTCAGCTTCTCCTCAAGCTTCAAGTAAAGGATTTGGGATTGTTCCTAAGAAGGCAGGGCTACTTAATGCGACATTACCACATGATATGCATATCTACCTCTGAAAACTGTTTCCATGGCTTTCAATCACATTGTACTATAAATCTAAGGGAAGAAAAGTGCACGCTTTGACTTTGAATTCATTTGTTGACGCTTAGTACATCAATGGCGTGATCTGCTGCATACTGCAAGTTGGATGCAACTTGTAAGAGCTAGAACACGATGGTAGACTGTCAAGCTCTTTGAATGGGTTTTGCCCTGTGACCATGGAAAGCTAGAGCGAGAGAGAGAGAGAGAGAGATGACGGTTGGAGAGGAGCCATCACACTATCCAACACAATTAGAGGGTGGGCATAAAAAAGGCCATGTGGGCCGCGAAAAGAGACCTCTCCTTCCCTCCCCCATCGCGTCGCGGACGGACGGGATCGTCCTAATGTCGTGATCGACGGCCCACATACTCACCCGAGATCGACCGACGGGGCCCACGCCGCGAGGGGAATGGTGGGAAGCTCACGTGGGAGAAACATGCGCTGCCGGCCACCTCATCTTCATCATCAACAGAAACGAGTGGAAGCCCACCCGCTGACATGGCACAATCGCAGCGGGCTCGATCGATACAAGTCAGGCCGCGCTCTGCGGCCGAAAGCCATCCCATGTGCTGTGCAGTGCCGTGAACACCCCTATTGTATCCCTTTCTTCCTTCGCATGTGTAGTCACAGTAACAAGATCATGACTCCGAATCCATGGAGCTCCTGCATCTTGGACGAGTTTGGTGTACCCAAAGAGGACGCATTGATGAGCGTGCCGAGATCCCAGGTATTGGTCATGGCAATTACCCCCATTCACCTTTGCTAGCAGAGTAATAACCGTGAATCGGATGCTAAGTTGCTTAGAGACTCTTGGGACTTCATCAATGCTACGGGACCAGATTGAGCCACAGCACCAACCTGCGCTTCTCTCTGGAACAAGGCAGTGGTTGGAATGCGCAAACGCCAGCAGTTCAATCTTTCGCTGCACTGTCTGCGTCCACAATCCATTCTGGGGGTTTTCAACTCTGCCTGACACTCGCCATGTGAGGGCTAAAGTTGAGACACCAGCAACAATAACTGTTCTCCGTTCCTCGATAACTTAGGGTTCTCATTCATTAATGTTCTGGGCAATTCAATTCGCCTCCATGCCCTCTTTCAGCCCTGAGACAGAGTCTTCTCTGATGTTGTACTACCAAAGCTTACTGTAGTATCATGTCTCTGGTTGTTAAGAAGACAATAGAACAGACATGGCAATGGGAGGTAATGTATATATGCATCTGAATAATTATTCTCTCAGTGTTTTGTTCTTGAGGGCATGGTGGTTTGGTAGTCAAATCTACCAAAAGATGAACGGAGAGGAACAGAGTATGTGTGGAGAAAGGTGCGTCACGTTTCCCATTATCTTTAGCTTCTAAGATTCCCCAAATCACGTACCTTTTTGGACCCCACCGGCATAGATTGTTCACACGACTTCCTCTCTCTTCCTTCTCTCATCCATAGCATCCTCTGCCTTTAAATCCCTTCGTTCCTCTTCGCATTACTCACAACTCTCAACTTACCGGAGAACAAAAATG

**>MaGAL (Ma07_g08800) promoter**

ATCAGTTTCTCTTGTTTCTTTGATGGGGTCTTCATCACTCCATGCAGTAGCGTGAAGAAGTTCTTCATCTATCTGTGCAACCTCGGCTGCCAAATATCATGGTCACATCAGAAGATGATTCCCCTTTAATTGAAACCAAGACGAACCTAAACAAAGACAGTTAATTATGTGCCAAATCGCTTCAGCTCAGTTCCACAATGATGTTCTCTTCTTCAGATTGGAGTCAGAGAAAGAAGATGAACTGTAATGGTAGTTGAGACTGCTGCCTCTCGACTAAGGTGACTACATGATGACAGATAAGATGGCACTAGGATCCTAAAAATCAGGTAGGGAGACCAAATTGCTTGTGTGTGTTTGGTAGATAAATAGTATGTGGATAGAGTTGTTAATGCACCCCCAAGTTGAGGGAGGTGATTTAGGGGCATGTCTCTTGCAAGGTCAGTATTTATAATGAGATTCCCTCACCCAAACCAAATGGAACCAACAAGAACATGGAATGTGAGGAAAATAATAGGTTATTAACAATATGAAAGATTAATCTAATGAAGGTCTGATCCCTCCATCCTTCATTACATTTATCTTCCAGATCAATCTAATTGGCTAAGAAACAACAAAACACATGTTATCTAACCCATTCAAAAAGTCTGATGACACAATGAATTCATCCAGCAATCTCTTTCCAGAGTTTGATGCGGGTGCCACATACTCTGAAACAATGAGTCACGCAAGAGAACTGGTAGGAGGAATACTACTACTACTACTACAGCTTTTGTGGTGGCTGTTGTTAGTCCATATGGGTAGGCTCAGTTGGTGCAATGTCTTCTTCAAGACTTTGATGCAGGTGCCCATGCATGCAAGGTCTAAAGCTGTACCTAAAAGAGCTGTAATCCTATGGAAGAAACTGATGACCAAAGCTTGAAACAATGAGCATGATAAAGAAGAACAAGATGCCTCCTTAACTGACCAAACTAAACCTCTCACAAAGAGATTAAACTAAACTCTCGCATGTTACTTTCACTGACAAAGCTAACCTTTTCTACCCTTCTTTTTCATGCAGGAGGAAGCAGAGCACGGGTTGGAGTACACTCCTGCTGCGGCTCGATGGTGTGTTGCTTCCTCATTCAATGGGAGTTGCTGAAGCATTTGAGGTGTGTGTGGTTGGTCAGTACATGCGTGCAGTTCCCTTCTTTTCTGGGTGGGATTTAACAGCCTACGTATGGGTTGCAGGGGTGGCAGCTCCTTTGGGGTTCAAATGTGTTTGAATTCGCGGGAGGACTGCTGCTGCTGCTGCTCACGGCCACAAGAAACAGAGGAAAAGAACAAGCTCTCGTACCTTTGCATGCTCGACGGTTCCTTGTGGCGTCTGGTTCGAAGTCTCAATGTTGCAGTGGAACGGAAGAGCCATGAGAGTTGTTGAAAGGATAATTAAATTATTAGTTTTGTTTAGCTTAAACCACTATTTAGCCTCAGGATTAGAGTAGTGTGCTCATCAGAGAGAGTCCAAACTTGATGGCACGCAGATCCCTAACTCCCAATGTGTATTGACCTCATCTGAGGTAATCACATGTAAAATTCTACCCATCTTTTTCCATGTGATTTTTCTTTTTTAGGTGCAAGAAAATTCCTGATAAGCTGTTCACGGGGAACGGAGGAAAACGTGAGTCAAGCATCCACGTTAGTGGGTGGGGCTGTGGGGTCCTCATGTTCCGGCGCCAATCGTGTTCTCGCCTTCGGCCCTCGGGTATTAAACCGCAAAGGCACCACCCCCTGCTCCCGCTCCACAGCCCCCCCATCACTCCCTCTTCCCTCCCTCCACTTTGATCTCCCTCTTTCTTCCTCGGTGTGTGCTTGTTACAGTGCCGCTACTGTTCAATAGTTCCACCAAATCTTCTCTAGCAGAGGAGAAGACGGACGAACAGAGCGCACCGATCTATCATACATAGATTATACGGCCAGGACACTAAAGGAGACTCCTTTGAAACTAAGACCGACCGGCCACCAAGGGCCTCGAGCCTATG

**>MaPG3 (Ma02_g04450) promoter**

CCCAGATGGAGTGGATTACCTAGAGAAAGACAAACTGTGAACATGATGTTGCCTGTAACATTTTGGTTTGGCATTGCAATAACTTAGGACAGTAACATGGCCATGTGATATTGGTAGTACTTGTGTACCTCAAAACCTTTGTGCAAATTGTAATGACAACATCCATGTTCATGCCTCTGATGTTTTTGACTGACAGCTGTGTTGCTTGACATCTTATTCGATGCTCTCTATTTTTTCATGAAGAATAGTTCATAACAAGTTGCTTTATTCCTCATGATGGGTCTCTCTTTAAGACTTTTTTTGCCTTACTGGAAGGGTTTGTGTTGTTCAGCTAATGTGCTCTTTTTCCAAGCAGTGATTTATTTCCTATCTTTATCTACCTAATCTCTCATCCTTTCTGCCTAGCCATCCTTACACCACCATATGAACCATTCCTACTGAATTTATTGGAAATAAATGACATGGTACTGAATCTAGTGAGAGGCCCAACATCCCTCCTTAAAGGATGAGGTATCACAAGGAACACTCTTGGCTTATTTTCTGTAATAAATTCTTCTTATTTGATTTCTTCATGGCATGGACAGGTAAAGATATGAGGGTCCTTCTCAAGGCCAACATTGAGCTCAGAATGCCATTTTATATTCATTAGGAGGGACAGTTGAAAGAAGGCCACCAAAATTACCAAGAAAAAAGAGGCATCCAACAACTTTCTCCACCAACCTTGCTTGCATACTCCACCATCTGGTCATTATCATGCTGCTACCACCAGGTGATGTCCGTTTCTTTGGCTTCTTATGCATCCACATCACCTCAGAAGTTTGAGGCCAAATGACCATTGAATAAATGAAATTAGCACTTGATTATTAAAGGATATGACAGGAGGTTGCATTGTTCATAGGGCTTATATGGGAAATGCATCAGTGAGGCATGGATTTGGTTGCATGAATCCATCAATTGGTAGGATGAGCTTAGACAGGCATCATAGGGGGACCTAAATGCAGTTAGAAAAGGTCAGGTTGGGATTTGGAAAGATGAGCTAACAGAGCTCTTCTACCAGCCTTTTCCTCAATAAGAGGAAACATGAGATCTATCTTTTGCCTGATAAAATATGAAAGTTCTTTGTAGGCAAATGATGCATCGTTTTCTGGAAAATATAGCAAGCAGTTCTTTAAGGTGTAAAAAAGCTTTGTTGATATTAAACATCACACAGACCAGCCAAAGTCAAAGATTAAGCAGCAGGGAGGTGTCATGTGTACCAAAACCCATCAATTTGTTCCAATAAAAAAGCTTGGATGAATGTGCTATTGGTTTTAGAGGCTCAAAGTCTCATTTGTAGGAGAATAAAATGCTGCTACTTTTCAGCTTTTGGAGCCCCAATCCATGTCCAAAAGAACAACAAAAAGAAAAGAGAACAGAAGAGAGAAAAAGATGCTCCAGAATGCAAAGCTATTGATTTACAATATTCTGTACATTAGAAGGACCCAATTGATCCATCCACTGTGGTGCAAAGCCATCAAGTGGAAGCTTGGTATCAGAGACAAAAGATAAAGTGATGGAGTATACCAGCTGAGCTTTTTCCAGAAGGAAATCCCACAGAACCTGTGAGGTTTCTTGTCTGCTTCTTCACTCTTTGATCAGCACAAGGATCCCAAGTTTAGATGACCAGAAAGCACAAAGATGGGAAGACTGAAAGAAAAGTAAGAAGTGGGTAGAAAGAAGGGTTGGAATACCACAGCGCCAAAGCAACTTTTCTATGCCCACCTTAGACATCTTTAGAGCGTATCACATGCGTCCACGCCTGCTCATGTTCTTCTCTTCGCTCAGCACATCTCCACCCGTCCGCTTCCCATCCAATGCACGGTTTGTAAGACTCCCATGCACTCTTGCTTCTTCCGGAAGAATTCCTCGTATAAAGAGGTAGAGGGAGAGGCTCCTCACCACCCTTCCTCTTCTCCTCTCCTCTCGTTTCAAGGCGCACAAGGGGACGAGAAAGAGTGGAGAGAAGGTGGGTCTGAGGAGATCATAGCTGATAACGATG

**>MaMYB69 (Ma04_g16770) promoter**

GTTGACGGTAAGGAAGACCATGTTTGCTTGTTAAAGAATTTCTTGTATGGATTGAAGCAGTTTCCAAGATAATGGTATAAGAGGTTTGATTCTTTTATGTTGGGTCATGGTTACAAGAGGAACATGTATGATAGTTGTGTCTACTTCCAGAAGTTAACTGATGGCTCTTTTGTGTATTTATTGCTTTATGTTGATGACATGTTTATTGCAGCTAAGAATTTGTCAAAAATTCACACTTTGAAAATGCAGCTGAGTAGTGAATTTGAAATGAAAGATTTGGGAGCAGCTAAGAAAATTCTTGGCATGGAGATCAAAAGAGAACGAGGAGTTGAAAATGATTTCTGACCCAAAATAATTACCACGAGAAAGTCTTGGAGAGGTTTGGCTTGAAAAACGCTAAGCCAGTGAGTACCCCTCTTGCTAGCCATTTTCGGCTATCTGTTGCTCAGTCACCACAGTTAGTTAAAGAGGAAGAATATATGGTGCAAGTTCCATATTTCAGTGCCATCGGTAGTATTATGTATGTAATGGTTTGTACTTATCCAAATATTTCACAAGCAATCAGTGTGGTTAGCAGATATATGTCTTGCCCGGGTAAAAAACATTGGCAGGCTATGAAGTGGATTCTCAGATACTTGCAAGGGACTTCAGATACTTGTTTGGAGTTTGGGAAAAATCGTGACACTTTGGTTGGTTTCGTCGACTCTGATTATGCTACGGATCTTGATAAATGAAGATCTTTGACAGGCTATGTTTTTTGCATTGGCGGTTGTGCAGTTAGTTGGAAAGCTTCCTTACAACCTATCGTGGTTTTATCTACTACAGAGACAAAATATATGGCAGTGATAGAGGCGATCAAAGAAGTTTTATAGTTAAGAGGATTATTCGACGAATTATATCTACATCAAGTTGTTACTACAATTTACTGTGATAGTCAAAGTGTTATTCATTTGACTAAAGACCAGATGTATTATGAGAGGACGAAACACATCGATGTGAAGTTTCATTTTATTCGGGATACCACTATTGAGGAAAAGGTCCTTGTTTAAAAAATTTATACGAAGGACAATCCAACTGATATGCTTACGAAGCCTCTTTCGGTTTACAAGTTCAAGTAGTGCTTGGACTTGGTTAGTGTTCATTGTTGGTGATTGCCCGTTGGGGCTTTTGTGAAGGTGGAGCAAGTTTTGTTAATTGTCAATGTTGGGACATGTCAAGGTGGAGATTTGTTAGTTTGACAAGTCCCACATAGTCCCACATCGAAAAAATCAAGCTCGTTATCTCTATTGCAACTATAAATAAGAGCCTAGCCTTGAAGCCTAATGCACCACAAGAAAAATCATTTGCTTTAGGTTTAATTTCTATTAAATCCACACTTAGTTAAAATAGTTTGGGCTTATAGTTTGAGATTTTCTTGTTTAATAGTTTTAGGTGTTTTATGGCTTACGAATATTTTCCTAAGGCATTTGTAATTGTCTCTTTTATAGTAAAAACATTTGTAATTTTCTGCTTTATTATCTTTATTGGATTGTCAAATTACCCATGATAAATTTTCTGGGACCAGCTCTAACATATAGTCTTATATTTATCATAAATAGAGATAGCAACATATATATATATATATATATAATGTACAGGATTCAAGAGTGGCATATAGAAGCTCTAATTTGCTCTAAGAGAGAGAGAGAGAGAGAGAGAGAGAGAGAGAGAGAGAGGTGGAAGACGACGTCCCCCACCTTTGCTTCCTCGCTTGGGGAAACGCTACTGCTTATTACCACTGTTCACTGATAAGTATTTGACAGGTGCCATGCCGCACACCTAAACCTCTTCTGCCCTTTTGATGGCGTGTCGTGTCTGCTAAGGTGCAGAAAGAGACCCCCTTCCATTGTATCGCAGGCGTCTACGTAGCTTTTGGAGGCTGTCTGCATGCGTCTCCACAATATCTGTAGATTTTGGAGGCTAAACGCTGCTATAAAACCCTCTGCCCGCCACCTCATGCCTCTTCTTTTTGTAGGTTGGAGGAAGGATCGATG

**>MaACO1 (Ma07_g19730) promoter**

GAAGCAAAGCCATTCTTTCCATATCACTTTACACGGCTAAAGCACGCAGTCTAATGGATGATGAAAGGAACCTAACCACTCTTACTGCACTGCATGTGAATGGTCACGCATCAAAGCAGGCCAGAGAGGAAGAATAAGATGCAGCAATGGAGTTCTTTCCCCGGCTGGTGTGTGATACAACTTCAGTCCCAGGAAAAGCAGAGGATGCAGAGCAAGCTGTTCTTCTTCCCTGAGTCGTAAAGCTTCTTTCACGCTACTGTAGTGTCTCTGTTGACAAAAGGTTCTTTGGCATTAGGAAGATGTAGAGCTTGACTTCTAGTTTGGGAGTTTGTGCCCTGGTACAGGAATCTTCTCACTGTGGTGGTGAACCAGTGAGTGAAGTCGATGTTAAATGCTACCGTCGACAGTGTGATCGTGTGTCTCAAGTGGGTTTAACTTGATGATTCGAGCTGGCTTGCTGCACTTGGTTTTCTTACAGTACGTGTGTGTCTCACAGACATAAACATGACTCTGTTGACAACAGGGTGAATTGGCATCATCATCCTTTACCTGTCCCTGATTTGAGCTGTAGTGCTGCGCTCAGCCAGCGTTTTATCGAAGAAGATAAACATGCGTGAGCCTACATGCACCAAAGCTTGCCGGCAAGTCATGCATAGCTGCAAACATGTGACAGGCACCGAACCAACAATTGAAGAAGATACGATAAACATGCGTGAGCCTACATGCACCAAAGCTTGCCGACAAGTCATGTTTGGGTGCACAATGTGTCCTCATCTTACTTGCATATCTGCTGTTGCACAACAGCAGATTGCATGGAGGTGTGTTTTCCGGCAATGCAATCTTTGATGTTGGTTCTCTTTTCTCTCTTCTTGCATTGTTTATAGCTCTGTTTCTTGTGCTCTTCTTTTACGTAGATTCATAGCGTAGCTTAAGTTGTTATAGATTACCTGTTTTACTGGGCAAACTTGTGCAACCCAGGAATATTCCCATGTGCATCTTCTTCCTGTTTTCCTCTGTCAAACTGTTCTGTTCATGATGAGGCAGCACCGAATCTAAGAGAAATATCCTAATGTTGATTGATTTAACCTCATAAAACTTGAAGCAGAATATGCTTGCCGCTTTCATGTGATCAATTGAATTGTTTGCTTGCTTCACGAGAACACCACATTCTGAACCCATTGCTTTCTTGTGGCCACCAACCGGAGAAAGGGAGTCTATATAACTAGCCGAGCGAGGATTTTCCCATGACCTGTTCATCTCACGTAGAGATGGTGATTTGGTTATAGTTATAGCGATCTATGATCGAAGAATGAGAAAATACCCAGATAACGGAGATCCATGCGTCACCAGATGGAACCTCGGCCGAGTGCGGCCGAGTGACACTGTTTGCACACCGGATACTTCATGTTCACGGCAATGGCCGACATGCCGAACAGCCATCGAGCGTTGAATGTAAGGCAGGAATGGCCCATTTCTCACATACGAGAGGGATACGAGTGGAAAGGGCGCTCTAATGAGCTGTGAATCGAAACAATTTCTACCTATCGATCCCTGTTCTTTTGATATGAAGTATAGCCAACAGGTCAAGAGAAGACGAGTACACACGCATCGCCGATGCTGTGACGTTACTTTCTGAGGTTGGCAATTTGTCACTACAATCCAAGCGGAAGCCATGCACGCGAGGCGTCGCCATGGAAGAACTCAACAACATGATGCCTTCCCGGGTCTCCTCAAAGGGGAGAGACCGATGGAAGCAGCCAAACTTGGTCCCCGATCGTGATGGGACGCGAGAGGTGGAAGCAAGGAGGGTGGAGAACCAGGCCAAAGGTGGTGGGGCTGAGAGATGGCCAACTGGGTCACCTTATGGAATCGGCTCCGTTACGTCTTCCACTGCTGTTGCTCTCGTCGATAGATCCTTCTCCAACTTTGCTTCTTCACTCATTTCGTCCCTCGACGTCAAGAACGCCTATAAATTGCCTGGTAATCAGCAGCACCTAGCACACTCCAGATAGAAAGCACAAGTGCAATCAGGGAAGAAAGAGCGTGTCATG

**>MaACS1 (Ma04_g35640) promoter**

GGTTTCACGGATCCGACGATGGCACCGAAAACCAAGAAAGAAATGGGGATGTCCAATCTAAAACGTTGCCGACATCTTAATCGTGCCTGAGATCATATATTACCTACAAACACACGTTATTTATTCTGGCTTTCAGTGGAATAATTACTCTTTACTTTCTTTGATGGATTGCTGGAAATGGATTGTTTGGATTGCGAACGAGAATATAAGAACAACATTCTACTTCAAATATTAATTTCACTTTTGATTCTCGGAAACTACACCCCATAAATTTGATCTTCGAAGCCTAAGAATTAAATCTAAATAATGGTAGAAGCATAATTTATTTCAGGTAGGTGGATGGCTTACATGCTCACCTAATAATTTATTTAAGTGGGACCCATGAATAGGCCACGTTACATTCAAAATTTGATGATTAGTGTACCATGATGAGCTGTGTAATGCGTGTCTTGATTGCGACTTCCCGTAGACCCCATCGATATTAGATTGAGACGGAAATAACGTCGGTCGGTAGACAACATAGTACGTAGATCATGATAGAACAATTCGAAATAATAAGACATACCAAAAAATTTGGTATACGACTCGATATATAAAAGTTAAAGTTATTTCTTTCATCTCTATATATATATACACAATTTGTAAGTTTCCTAAAAGATATTAGATTTTGGGTCAACTATAATTGCCTTCCAACAAAGTAGAATGCCTCTATCAGAATCTCGCGCCTACAAACAACACCAATCCTAATCATTCACATGATCTTAACGTTAATGAAGTAGCAAATTCCAAATCACATTTAAATAGCCAGTCATTTCAAACATGAAGATCATGCTTTTATTGTTTCTTTTTTGGTAGAAGAGTGAATGAGATCCACATTTTCCTTAAGAGGATGCATATAAGCCAATTAAATTAACATCCATATGTATAAAATCCAATTAGGCAAAGGCAAATCGAAGTAGTCAGATACGTTTCTATCTGATCCGATTAATATTCTCTTTTACGATTGATAAAGAATACATGCTAATTGATATGGGAGGCAATTCCCGACGTATCATACTCATCCGATCGATACGTAATATCGTTAGGTCAGTGATGAAGATGTTAATTTGTCTCACGAGATCGAAATATATAATTAGAAAAGTTGACATGACAAGTCAGTTATTCTATAACCATAGTTTTGATTTCATGACTCATCGTGACATTTATTATCGTCTTAATTTAATTATCATATATTATAATATAAAAGGTGTTCTAGTGGGAGTTTTGACTTAATACATTATTTAATTTTAATGCTTGATTGAATTCTTCAAACTTTTACTAACCTAAATATTGACTAAATAAGTATGCTTCGACAAGATTTTTTCTACGTGATTAAATCTTTGACCTCTTAAACACCTCAACTTGAGTCAAACTCGGAAACTCGAAAGTGGACCTCCGTCAGACTCCTTTAACATCTGTTAAACAACTTGAATCTAATTTGACACTAATCAAAATCAGACTGCATTGAGCCTAAATGGATCTAAATATTAAGAAAACCATTGTTGAATTTTCCTTCTTTCGCAAACAGCATGTCATCGATGAGATTAAGGTTTACAAAGAGCGGCGCACAATTTTGTTTTGGGATAAATAATTCTGTGCTTACAATATAGAAGAGTTCGAGTCGAAAGCGACTCCCGAGTTCGGAACACGTCATTGTTGCCGCCAACACTGAAGCTTCCTATTTGGCGTCACCTGTCGATGTTACGGCGCATCCATCGCCAATCACGTCCATGATTTACACGCTGCCGGATCGACTCGGTTTTCATGTCCTTCTTTTCCAGCCTGAAGTCCTCTTTGTTGACCTCTTTGGATGTTTGAATGGTCTCGGGATTTGCCTATTAATGGTCATCGGAATCGACTCTTGCAAACTGCAGCAGCTGCTTCTCCTTCTTCTTCTCTGCTCGCTTCAGCCTTTTCCGGTACGTACCTGAGATAACGGGTCACATG
